# Expanding the Functions of KHSRP Protein: Insights into DNA G‐Quadruplex Binding

**DOI:** 10.1002/advs.202410086

**Published:** 2025-01-06

**Authors:** Pasquale Russomanno, Pasquale Zizza, Linda Cerofolini, Federica D'Aria, Sara Iachettini, Serena Di Vito, Annamaria Biroccio, Jussara Amato, Marco Fragai, Bruno Pagano

**Affiliations:** ^1^ Department of Pharmacy University of Naples Federico II Naples 80131 Italy; ^2^ CERM‐CIRMMP and Department of Chemistry “Ugo Schiff” University of Florence Sesto Fiorentino (FI) 50019 Italy; ^3^ Translational Oncology Research Unit IRCCS‐Regina Elena National Cancer Institute Rome 00144 Italy

**Keywords:** biophysical methods, gene promoters, G‐quadruplex DNA, protein‐DNA interactions

## Abstract

KHSRP (KH‐type splicing regulatory protein) is a multifunctional nucleic acid‐binding protein that regulates various cellular processes, with critical roles in controlling gene expression. G‐quadruplexes (G4s) are noncanonical nucleic acid structures involved in essential cellular activities, including gene expression, and are recognized as potential therapeutic targets in cancer. The biological functions of G4s are mediated by proteins making their formation highly dynamic within cells. Therefore, the recognition of G4s by specific proteins is crucial for modulating physiological and pathological pathways. Given the growing interest in DNA G4s, a deeper understanding of the proteins that interact with them and their molecular recognition is imperative. This study demonstrates that KHSRP binds to these DNA structures. Biophysical analyses provide insights into the thermodynamics, kinetics, and structural aspects of these interactions, showing that G4 structural variability significantly influences KHSRP binding, in which the KH3 protein domain plays a key role. Validation of these interactions in cancer cells further highlights their biological relevance. Notably, the G4 ligand pyridostatin affects KHSRP/G4 interactions both in vitro and in cells, suggesting that small molecules can modulate this molecular recognition. These findings underscore KHSRP's potential role in regulating cellular mechanisms through binding to G4‐forming DNA, positioning it as a possible therapeutic target in cancer.

## Graphical abstract

This study demonstrates that the KHSRP (KH‐type splicing regulatory protein) binds to G‐quadruplex DNA structures. In‐depth biophysical experiments reveal the energetics and molecular determinants of protein/DNA interaction. Biological analyses confirm these interactions in cancer cells, underscoring their biological significance. Ligand binding to G‐quadruplex DNA impairs KHSRP recognition, suggesting that small molecules can affect the protein binding process.

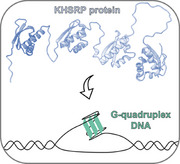

## 1 Introduction

KHSRP (KH‐type splicing regulatory protein), also known as KSRP or FUBP2 (far upstream element‐binding protein 2), is a multifunctional nucleic acid‐binding protein present in both the nucleus and cytoplasm, that has emerged as a multifaceted regulator of diverse cellular processes with critical roles in fine‐tuning gene expression.^[^ ^1^, ^2^ ^]^

**Figure 1:**
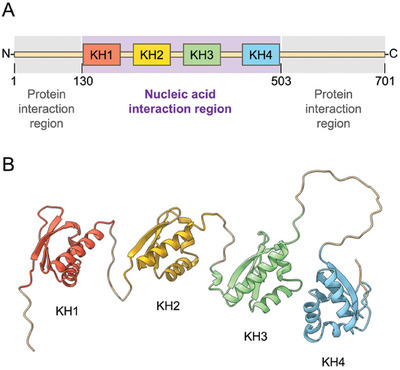
A) Domain organization of full‐length human KHSRP (KHSRP_fl_), and B) structural model of the central nucleic acid binding region consisting of the four KH domains (KHSRP_130‐503_) obtained with AlphaFold DB. The Protein Data Bank codes used for the four KH domains are 2opu (KH1), 2opv (KH2), 2hh3 (KH3), and 2hh2 (KH4).

KHSRP protein structure is organized in three distinct regions: two N‐ and C‐terminal low sequence complexity regions that contain several sites for post‐translational modifications as well as protein‐interaction motifs, and a mainly structured central region comprising four hnRNPK homology (KH) domains responsible for nucleic acid binding (**Figure** **1**).^[^ ^3^ ^]^ The modular structure of KHSRP and its consequent binding flexibility enables the protein to interact with multiple targets.^[^ ^1^ ^]^

Originally identified as a factor interacting with an enhancer element upstream of the *c‐MYC* oncogene promoter thereby stimulating its expression,^[^ ^4^ ^]^ KHSRP has since been implicated in a plethora of post‐transcriptional processes, highlighting its functional diversity and versatility.^[^ ^5^, ^6^ ^]^ The RNA‐binding capability of KHSRP allows it to interact with a wide range of RNA species, including messenger RNAs (mRNAs), non‐coding RNAs, and viral RNAs.^[^ ^1^, ^5^, ^6^, ^7^, ^8^ ^]^ Moreover, KHSRP has been shown to participate in alternative splicing regulation, contributing to the generation of transcriptomic diversity and the production of functionally distinct protein isoforms.^[^ ^5^, ^6^, ^9^, ^10^ ^]^ The multifaceted functions of KHSRP are not limited to RNA metabolism but also extend to broader cellular processes. Indeed, KHSRP has been implicated in cell proliferation, apoptosis, development, and neuronal processes.^[^ ^5^, ^10^, ^11^, ^12^, ^13^ ^]^ For example, it was recently shown that KHSRP controls monocytic differentiation by interacting with specific genomic sites in promoter and enhancer regions to regulate the expression of several genes through transcriptional activation and bind to pre‐mRNA intronic regions to modulate alternative splicing, thus showing the dual DNA‐ and RNA‐binding activity of the protein.^[^ ^14^ ^]^

Given its diverse functions, altered KHSRP expression has been associated with various human diseases, including cancer, cardiovascular diseases, and neurological disorders, underscoring its clinical relevance.^[^ ^5^, ^6^, ^15^, ^16^, ^17^ ^]^

G‐quadruplexes (G4s) are nucleic acid secondary structures that can arise under physiological conditions from guanine(G)‐rich sequences usually containing four or more consecutive G‐runs of at least two guanines.^[^ ^18^ ^]^ These structures consist of the stacking of at least two G‐tetrads, the planar association of four guanines held together by Hoogsteen hydrogen bonds. Therefore, they are markedly different from canonical double‐helical DNA.^[^ ^19^ ^]^ Moreover, G4s can exhibit a high degree of structural polymorphism: depending on the sequence, loop length, and base composition, the relative orientation of G‐runs can be parallel, antiparallel, or mixed (referred to as hybrid), giving rise to different conformational topologies. G4 folding and stability are strongly influenced by the presence of cations.^[^ ^20^ ^]^ Cation coordination is indeed required to form G4s, to stabilize the G‐tetrad stacks. Cation type and concentration can also significantly impact G4 conformations, an especially relevant factor given the polymorphic nature of these structures, potentially affecting their functional roles in biological systems.

It is well established that G4‐forming sequences are present in the human telomeric DNA and in the promoters of several oncogenes, such as *c‐MYC*, *c‐KIT*, and *BCL‐2*,^[^ ^21^ ^]^ playing important roles in various biological functions.^[^ ^22^ ^]^ In the cellular environment, the formation of G4 structures is heavily influenced by interactions with a multitude of protein factors.^[^ ^23^, ^24^, ^25^, ^26^ ^]^ These protein interactors can alter the equilibrium between unfolded G‐rich sequences and folded G4 structures, either stabilizing or destabilizing them, which can impact gene expression. As a result, the formation of G4 structures in living cells exhibits remarkable dynamism. Interestingly, numerous single‐stranded RNA‐binding proteins are known to directly interact with G4s.^[^ ^27^, ^28^, ^29^, ^30^, ^31^ ^]^

Biochemical studies conducted more than 20 years ago revealed that KHSRP is a component of a multiprotein complex that includes heterogeneous nuclear ribonucleoprotein F/H1 (hnRNPF/hnRNPH1) and polypyrimidine tract binding protein (PTBP1),^[^ ^10^, ^32^ ^]^ with the first two more recently shown to interact also with G4 structures formed by RNA sequences.^[^ ^27^, ^33^, ^34^ ^]^ It has also been observed that KHSRP can associate with hnRNPA1 in different cell types and that the two proteins show similar binding preferences to RNA sequences,^[^ ^35^, ^36^ ^]^ with a preferred binding target for both proteins including G‐rich sequences.^[^ ^11^, ^36^, ^37^ ^]^ KHSRP and hnRNPA1 have also been proposed to have antagonistic roles in the post‐transcriptional regulation of miRNA expression.^[^ ^36^ ^]^ According to the literature reports, hnRNPA1 also participates in the resolution of G4 DNA structures at the end of telomeres to stimulate telomere elongation.^[^ ^38^, ^39^ ^]^ Furthermore, it is able to recognize and unfold G4s in some gene promoter regions, including those found in *KRAS* and *TRA2B* gene promoters, thereby modulating their transcription in cancer cells.^[^ ^40^, ^41^, ^42^ ^]^ Indeed, accumulating evidence indicates that G4 DNA formed by promoter sequences is involved in the regulation of gene expression.^[^ ^23^, ^43^, ^44^, ^45^ ^]^

In a previous chemoproteomic‐driven study, some of us identified KHSRP as a potential interacting partner of human telomeric G4 DNA.^[^ ^46^ ^]^ Understanding the intricate functions and regulatory mechanisms of KHSRP is essential for unraveling the complexities of gene regulation. In this study, we demonstrated KHSRP's ability to interact with the G4‐forming sequences located in oncogene promoter regions. By integrating biophysical and biological analyses, we gained valuable insights into the energetic aspects and molecular characteristics of KHSRP binding to G4s from the *c‐MYC* and *c‐KIT* gene promoters, to which the protein showed a preference. Noteworthy, the validation of these interactions in the cellular context highlighted their biological relevance. Our findings shed light on the involvement of protein domains in recognizing these DNA structures, as well as on the G4 structural determinants that contribute to this interaction.

## 2 Results

### 2.1 Protein Expression, Purification, and Assignment of Protein Residues

The full‐length KHSRP protein (KHSRP_fl_, residues 1–711, Figure S1, Supporting Information) contains a folded central region that includes the four KH domains (residues 130–503) responsible for nucleic acid binding,^[^ ^37^, ^47^, ^48^ ^]^ and two unfolded regions located at the N‐ and C‐terminus (1–129 and 504–711, respectively) (Figure 1). So far, eukaryotic systems have been utilized to express KHSRP_fl_, followed by protein purification using the ammonium sulfate precipitation method and subsequent refolding.^[^ ^10^, ^49^, ^50^ ^]^ Here, we have devised an alternative approach to obtain the protein in its native folding, consisting of using *Escherichia coli* as the expression system, and a purification method based on a tandem combination of GST affinity and size‐exclusion chromatography (see Figure S2 for protein construct, Supporting Information). The identity and purity of KHSRP_fl_ were ascertained by SDS‐PAGE analysis and 1D ^1^H‐NMR spectroscopy (Figure S3, Supporting Information). The NMR spectrum of KHSRP_fl_ obtained through the tandem purification method was compared with that of the protein purified exclusively by GST affinity chromatography. Both purification methods successfully yielded properly folded protein. However, only the tandem method allowed for obtaining the protein completely free of bacterial DNA, as evidenced by the absence of signals in the 5–6 ppm region of the spectrum, which corresponds to the resonances of DNA sugar protons (Figure S3, Supporting Information). However, due to the full‐length protein's low stability and high spectral complexity, its utility in the study of the protein‐G4 interaction was limited. Consequently, we opted to proceed with the investigation using the truncated form of the protein (KHSRP_130‐503_) comprising solely the four KH domains (see Figure S2 for protein construct, Supporting Information). The expression and purification of the KHSRP_130‐503_ protein were conducted using the procedures described by Gherzi et al.^[^ ^49^ ^]^ and Kung et al.^[^ ^50^ ^]^ with slight modifications. These modifications included the implementation of protein overexpression in various *Escherichia coli* strains and the optimization of the protein solubility by using a low growth temperature (see the Experimental Section for details, Supporting Information). SDS‐PAGE and 1D ^1^H‐NMR analysis confirmed the identity and purity of KHSRP_130‐503_ (Figure S4, Supporting Information).

The spreading of the signals in the 2D ^1^H‐^15^N HSQC spectrum acquired on [U‐^15^N] KHSRP_130‐503_ is consistent with the presence of well‐structured protein domains (Figure S5, Supporting Information). Interestingly, the 2D ^1^H‐^15^N HSQC spectrum showed sharp and well‐resolved signals for a protein of 40 kDa, corroborating the presence of interdomain flexibility. This finding is also supported by the signals’ overlap observed in the central region of the spectrum since most of the residues of the linkers are expected to resonate at these frequencies. The backbone assignment of the protein has been obtained from the analysis of triple resonance spectra recorded on samples of [U‐^13^C, ^15^N] KHSRP_130‐503_ (see the Experimental Section and Figure S6, Supporting Information). ≈83% of the total expected non‐proline spin systems have been assigned (Figure S5, Supporting Information); most of the unassigned residues are in the loop regions, mainly in the long linker connecting KH3 and KH4 domains (Gln275‐Lys281, Arg320‐Gly324, Arg340‐Glu343, Ile386‐Leu390, Pro401‐Trp420). Twenty‐two proline residues, out of a total of 34, have been assigned by integrating the data obtained from the ^1^H‐ and ^13^C‐detected NMR spectra. The protein resonance assignment has now been reported in the BMRB database under the accession code: 52573. The prediction of the secondary structure elements, obtained by TALOS+ analysis using the resonances of KHSRP_130‐503_ as input (Figure S7, Supporting Information), is in agreement with the folding of protein reported in the AlphaFold structural model.^[^ ^51^ ^]^

### 2.2 NMR Investigation of KHSRP_130‐503_ Interaction with DNA G4s

1D ^1^H macromolecule‐based NMR experiments were performed as a first biophysical assay to detect the interaction between KHSRP_130‐503_ and several DNA G4s which differ in topology and/or loop length and composition and/or presence of flanking residues. In particular, three parallel‐stranded G4s, formed by the G‐rich sequences of *c‐KIT* (*c‐Kit1* and *c‐Kit2*) and *c‐MYC* (*c‐Myc*) oncogene promoters,^[^ ^52^, ^53^, ^54^ ^]^ and two hybrid topology G4s from the promoter region of the *BCL‐2* (*Bcl‐2*) oncogene and human telomeric DNA (*mTel_24_*),^[^ ^55^, ^56^ ^]^ were chosen for the investigation. Since we were dealing with two macromolecules, both protein‐based and DNA‐based assays have been performed, where changes in the line width of the ^1^H nuclei belonging to the protein or DNA have been analyzed, respectively.

Simple 1D ^1^H NMR experiments can be used to demonstrate the protein‐ligand interaction by observing signals in the aliphatic part of the protein's spectrum (typically below 0.7 ppm) both in the absence and presence of the potential binder.^[^ ^57^ ^]^ Indeed, changes in intensity and/or chemical shift of these signals provide immediate evidence of ligand binding, and qualitative information on its affinity by analyzing the evolution of the chemical shift of the signals upon the addition of increasing amounts of the investigated compound. Instead, for the experiments based on the observation of the signals of DNA, information on ligand binding can be obtained by monitoring protons resonating in specific regions, according to the specific DNA secondary structure. Concerning a G4 structure, the peaks of the imino protons assigned to the guanines involved in G‐tetrad formation, exhibit distinct chemical shifts typically ranging from 10.5 to 12 ppm.^[^ ^58^ ^]^ Analysis of the changes affecting these signals can be used to monitor structural alterations of G4 structures, as well as reveal interaction with ligands and proteins.

Therefore, 1D ^1^H NMR spectra were recorded on KHSRP_130‐503_ and on the different DNA G4s in the absence and presence of increasing amounts of the investigated G4s or the protein, respectively. As for the former, a significant decrease in the intensity of the proton signals of the protein located in the aliphatic region of the spectrum was observed upon the addition of G4s, clearly indicating the formation of the corresponding protein‐DNA complexes (Figure S8, Supporting Information). Interestingly, these variations were more significant for *c‐Kit1*, *c‐Kit2*, and *c‐Myc* (all G4s with parallel topology) than for *Bcl‐2* and *mTel_24_* (which adopt hybrid G4 conformations). Additional information on ligand binding can be obtained from the 1D ^1^H spectra by observing the region ≈10 ppm where NH proton signal of the Trp indole rings resonate and no signals of DNA are present. Indeed, if a Trp residue is close to the binding site, this region can be used to monitor ligand binding.^[^ ^57^ ^]^ Interestingly, we observed clear changes in chemical shift and/or intensity of such a signal when each of the investigated G4s was added. These changes varied thus suggesting different affinities of the DNA molecules for the protein or different binding modes.

**Figure 2:**
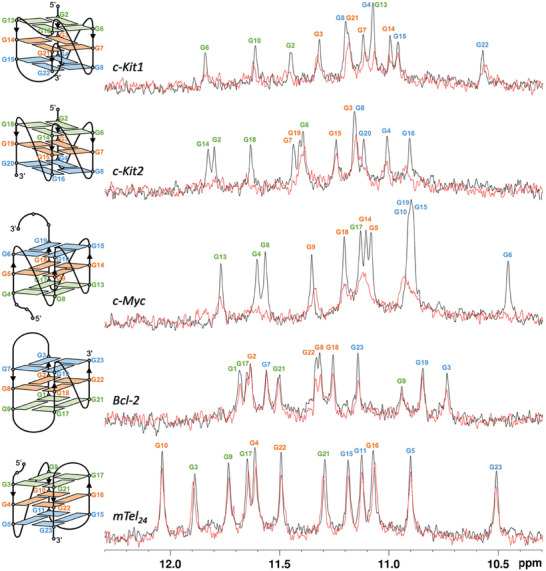
(Left) Schematic representation of the folding topology of the investigated G4s. The guanines forming the G‐tetrads are depicted as colored boxes. Strand directionalities are indicated by arrows. (Right) Imino proton spectra of G4s (20 µm) at 25 °C in the absence (black lines) and presence (red lines) of the KHSRP_130‐503_ protein (1:1 ratio).

Comparable results were obtained in DNA‐based experiments. A general decrease in the intensity of imino proton signals was observed for all G4s (**Figure** **2**), thus confirming the ability of KHSRP_130‐503_ to interact with all of them, albeit with a different affinity. Indeed, greater variations were observed for *c‐Kit1*, *c‐Kit2*, and *c‐Myc* G4s than for the others, again suggesting stronger interactions with parallel‐stranded than hybrid G4s. Furthermore, the DNA‐based experiments also provided insight into the G4 residues mostly affected by the interaction with the protein. For *c‐Kit1*, *c‐Kit2*, and *c‐Myc* G4s, the guanine peak intensities of the inner G‐tetrads resulted less altered than those of the outer G‐tetrads, which is consistent with external protein binding without structural changes to the G4s.

Moreover, in the case of *c‐Myc*, significant shifts were observed for the aromatic protons of adenine base (A12) belonging to the double nucleotide loop (Figure S9, Supporting Information), which is located toward the G‐tetrad groove pointing to the top of the G4 structure.^[^ ^54^ ^]^ This observation suggests that the interaction with the protein leads to the displacement of this base. These findings may also explain the minor effects observed in the interaction between the protein and hybrid G4s, where the external G‐tetrads are rather occupied by the loops, making them less prone to interact.

To better assess differences in protein binding to the external G‐tetrads, the data from the 1D NMR titrations of G4s with KHSRP_130‐503_ were further analyzed. Specifically, in each spectrum, the intensities of the imino proton signals were normalized relative to an isolated signal unaffected by the addition of KHSRP_130‐503_. Normalization was performed on the signal of G3 for *c‐Kit1*, G15 for *c‐Kit2*, G9 for *c‐Myc*, G7 for *Bcl‐2*, and G5 for *mTel_24_*, respectively. The normalized intensities of the signals in the presence of KHSRP_130‐503_ were compared to the normalized intensities of the same signals in the absence of the protein. The residues most affected by the interaction with KHSRP_130‐503_ (G2, G4‐G13, G6, and G10 for *c‐Kit1*; G2, G7, G14, and G18 for *c‐Kit2*; G4, G5, G6, and G8, for *c‐Myc*; G1 and G3 for *Bcl‐2*; G15 and G21 for *mTel_24_*) suggest different binding modes of KHSRP_130‐503_ for the G4s (Figure S10, Supporting Information). In particular, the G‐tetrad at the 5′‐end is primarily affected in *c‐Kit1* and *c‐Kit2*, whereas for *c‐Myc* the 5′‐end G‐tetrad together with a side of the G4 are mostly affected by protein binding. On the other hand, the interaction of KHSRP_130‐503_ with *Bcl‐2* and *mTel_24_* appears weaker and less specific.

### 2.3 Assessment of Binding Kinetics and Affinity of KHSRP_130‐503_ Toward G4s

Surface plasmon resonance (SPR) experiments were conducted to quantitatively assess the binding affinity of KHSRP_130‐503_ to the studied G4s and obtain insights into the kinetic parameters of the interaction. SPR is a powerful technique for real‐time investigation of DNA‐protein interactions as it allows measurement of the association (*k*_on_) and dissociation (*k*_off_) rate constants, enabling the determination of the equilibrium dissociation constant (*K*_d_) values.^[^ ^59^, ^60^, ^61^ ^]^

**Figure 3:**
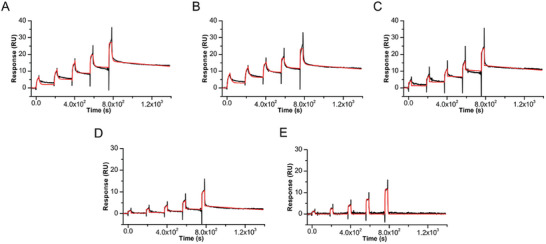
Time evolution SPR sensorgrams obtained at 25 °C by injections of various concentrations (from 1 to 20 µm) of A) *c‐Kit1*, B) *c‐Kit2*, C) *c‐Myc*, D) *Bcl‐2*, and E) *mTel_24_* G4s on the chip‐immobilized KHSRP_130‐503_, with a contact time of 30 s and a flow rate of 30 µL min^−1^. The sensorgrams are shown as black lines and their respective fits as red lines.

**Figure** **3** shows representative SPR sensorgrams obtained for the interaction of immobilized KHSRP_130‐503_ with increasing concentrations of the investigated G4s using the single‐cycle kinetics (SCK) method. Representative replicates for each DNA, along with their corresponding fits, are shown in Figure S11 (Supporting Information). SCK was chosen over multi‐cycle kinetics due to its advantage of using a single regeneration step at the end of the complete binding cycle. This single regeneration step is less detrimental, resulting in a longer lifespan for the chip surface and immobilized protein.

Almost all sensorgrams showed a response proportional to analyte concentration in solution, thus indicating the specificity of DNA/protein interactions. SPR data were then analyzed in the framework of the 1:1 binding model to derive the kinetic rate constants, from which the equilibrium dissociation constants were determined. The *K*_d_ values presented in **Table** **1** show a stronger interaction between the protein and the *c‐Kit1*, *c‐Kit2*, and *c‐Myc* G4s, as compared to the *Bcl‐2* and *mTel_24_* G4s. These findings are consistent with the NMR data.

**Table 1:** Kinetic parameters and dissociation constants for the interaction of the investigated G4s with KHSRP_130‐503_, determined by SPR.

G4	*k*_on_ [× 10^3^ M^−1^ s^−1^]	*k*_off_ [× 10^−4^ s^−1^]	*K*_d_ [nM]
*c‐Kit1*	3.1 (±0.6)	5.4 (±1.7)	190 (±90)
*c‐Kit2*	5.0 (±2.0)	2.8 (±1.5)	56 (±8)
*c‐Myc*	2.2 (±0.8)	2.8 (±1.5)	130 (±25)
*Bcl‐2*	0.8 (±0.5)	12.9 (±2.0)	2000 (±980)
*mTel_24_*	4.1 (±2.7)	50.9 (±12.8)	1470 (±480)

Noteworthy, protein binding exhibits a difference in affinity of approximately ten‐fold or more between parallel (*c‐Kit1*, *c‐Kit2*, and *c‐Myc*) and hybrid (*Bcl‐2* and *mTel_24_*) G4s. The comparison of the kinetic constants indicates that the disparity is primarily attributed to a variation in the dissociation rate rather than the association rate. In fact, the protein demonstrates a relatively similar association with the different G4s, while the complex dissociates more rapidly in the case of hybrid G4s compared to parallel ones. Among the parallel G4s, KHSRP_130‐503_ exhibited the highest affinity for *c‐Kit2*, followed by *c‐Myc*. In this case, the analysis of the kinetic constants indicates that the difference lies in the rate of association.

### 2.4 Circular Dichroism (CD) Spectroscopy Analysis of DNA G4s in the Presence of KHSRP_130‐503_

**Figure 4:**
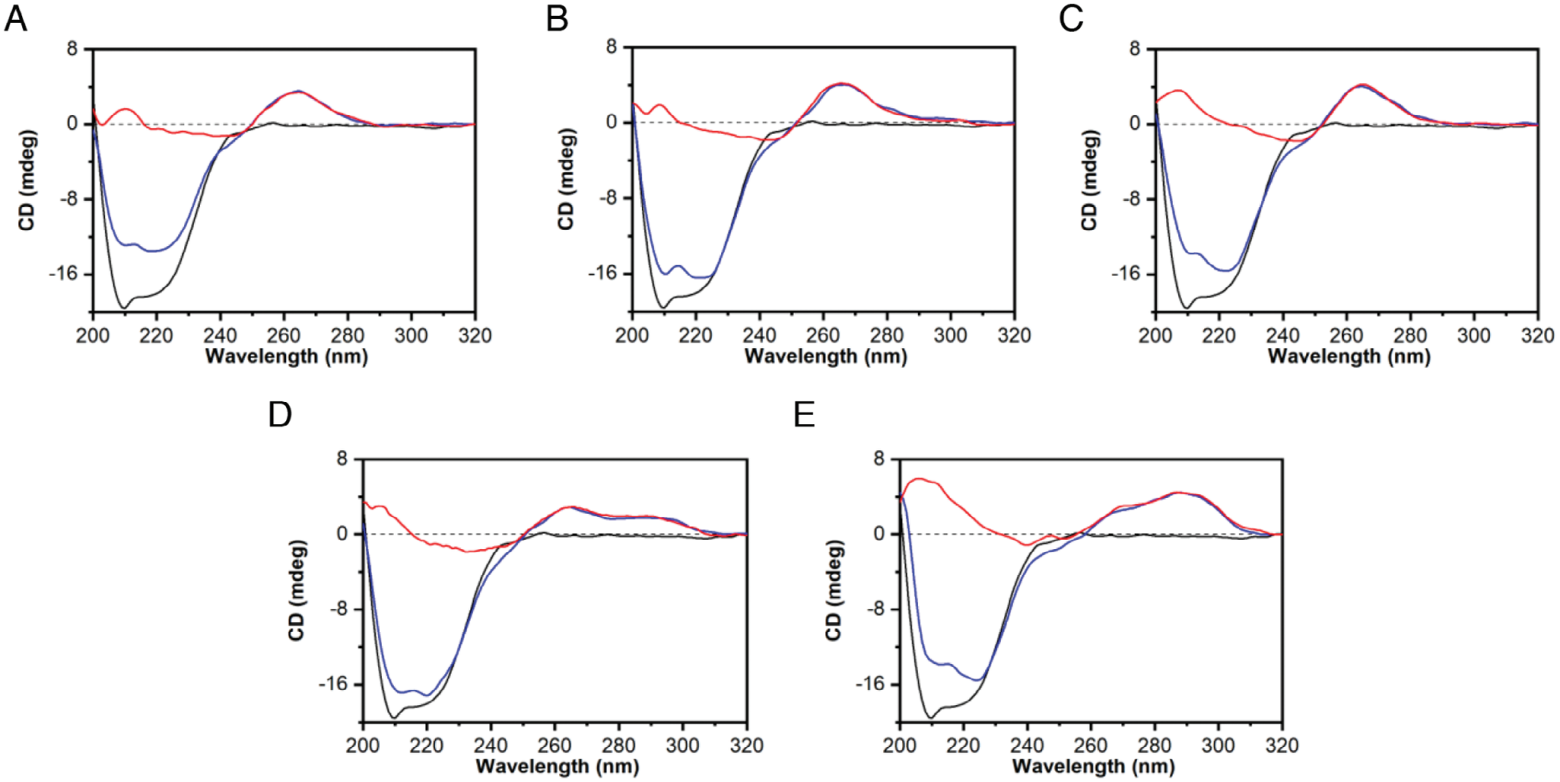
CD spectra of A) *c‐Kit1*, B) *c‐Kit2*, C) *c‐Myc*, D) *Bcl‐2*, and E) *mTel_24_* G4s in the absence (red lines) and presence (blue lines) of KHSRP_130‐503_ (1 molar equiv.) at 25 °C. The CD spectrum of the protein alone (black lines) is also shown in each panel for comparison.

To verify that the decrease in the intensity of the G4 imino protons observed in the DNA‐based NMR experiments was not caused by DNA structure unfolding or secondary structure changes, CD experiments were conducted. CD spectroscopy is extremely sensitive to conformational changes of nucleic acids and provides characteristic spectra depending on the structure adopted.^[^ ^62^ ^]^ Consistent with the presence of a parallel G4 topology, the CD spectra of *c‐Kit1*, *c‐Kit2*, and *c‐Myc* exhibited the typical signature of this structure, characterized by a positive band at 263 nm and a negative band ≈240 nm (**Figure** **4**).^[^ ^63^ ^]^ On the other hand, *Bcl‐2* and *mTel_24_* displayed CD spectra with two positive bands at ≈268 and 290 nm, along with a negative band ≈240 nm, indicative of hybrid‐type G4 topologies.^[^ ^63^ ^]^

The CD spectra of KHSRP_130‐503_ alone, as well as those of protein/G4 (1:1 molar equiv.) mixtures, were also recorded and are shown in Figure 4. Notably, no unfolding or structural alterations of DNA G4s were observed upon protein interaction. Indeed, the presence of KHSRP_130‐503_ does not significantly affect, both in terms of signal intensity and shape, the CD spectrum of any of the G4s at wavelengths above 245 nm, which corresponds to the spectral region where only the G4 chromophores absorb. However, it should be noted that for interactions with higher *K*_d_ values (i.e., *Bcl‐2* and *mTel_24_*), the complex is only partially formed under the assay conditions. These experiments were also conducted with protein concentrations two‐ and four‐fold higher than that of the G4s (Figure S12, Supporting Information). No significant perturbations in the structures of the G4s were observed, even in the presence of an excess of protein. This confirms that KHSRP_130‐503_ interacts with the G4s without affecting their structure, at least under these experimental conditions.

Furthermore, CD melting experiments were performed to assess any potential stabilizing or destabilizing effect of the protein on the studied G4 structures. Interestingly, distinct effects on the thermal stability of G4 structures were observed upon the addition of KHSRP_130‐503_ (Figure S13, Supporting Information). No changes were observed in the thermal stability of *c‐Kit1* and *Bcl‐2* G4s. On the other hand, the CD melting curves showed a slight decrease in the stability of *mTel_24_* (Δ*T*_m_ = −2.2 ± 0.5 °C) and a significative increase in the stability of *c‐Kit2* and *c‐Myc* G4s (with Δ*T*_m_ values ≈5.3 ± 1.0 °C) in the presence of the protein.

### 2.5 Structural Characterization of KHSRP_130‐503_ Interaction with *c‐Myc* and *c‐Kit2* DNA G4s

**Figure 5:**
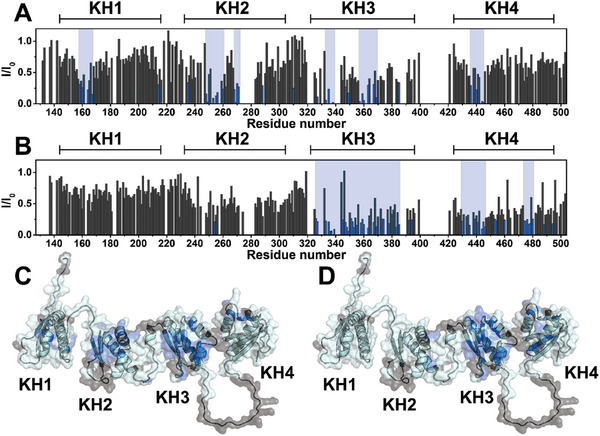
Graphical representation of the per‐residue intensity changes of KHSRP_130‐503_ (50 µm) in the presence of A,C) 25 µm *c‐Myc* and B,D) 6.25 µm *c‐Kit2*. The residues exhibiting the highest decreases in signal intensities (*c‐Myc*: Leu158, Ile160, Gly164, Ile167, Arg214, Val235, Val248, Ile249, Gly253, Glu254, Thr255, Ile256, Lys257, Gln260, Ile268, Leu269, Ile270, Gln271, Asp288, Gly310, Val327, Ser333, Val334, Gly335, Val336, Val337, Ile338, Gly339, Lys347, Gln349, Gln357, Phe358, Lys359, Gln360, Gly363, Glu367, Ile369, His371, Ile385, Cys436, Ile440, Gly444, Glu445; *c‐Kit2*: Leu247, Glu254, Asp326, Val327, Val329, Ser333, Val334, Gly335, Val336,Val337, Gly339, Ile345, Lys347, Ile348, Gln349, Asp351, Ala352, Gly353, Arg355, Gln357, Phe358, Lys359, Gln360, Gly363, Glu367, Ile369, Met373, Gly374, Arg378, Ala383, Ile385, Ser392, Arg394, Ser430, Lys435, Val439, Gly441, Gly444, Glu445, Asn446, Gln453, Thr454, Ile460, Leu474, Phe475, Ile476, Ile477, Arg478, Ser480, Lys488, Lys494) are colored in blue in the plots A,B) and on the AlphaFold model of the protein C,D). Residues that could not be assigned in the NMR spectra are colored in gray on the protein model.

Mapping of the surface of KHSRP_130‐503_ protein involved in the interaction with different G4 molecules has been obtained by monitoring changes in cross‐peak intensity (*I*/*I*_0_) and/or in chemical shift (Δδ), occurring in the 2D ^1^H–^15^N HSQC spectrum of the uniformly ^15^N‐isotopically enriched protein upon the addition of increasing amounts of the G4‐forming DNAs, versus the spectrum of the free protein in solution. In particular, the effect of the two parallel G4s showing the highest affinity for KHSRP_130‐503_, namely *c‐Myc* and *c‐Kit2*, was investigated and characterized. In the presence of both *c‐Myc* and *c‐Kit2*, we observed changes in signal intensity (Figure S14, Supporting Information). In the presence of low concentrations of G4s, largest effects are observed for *c‐Kit2*, suggesting a higher affinity of the protein for this G4. **Figure** **5** shows the plot of the per‐residue changes in signal intensity of the protein in the presence of each DNA molecule. Interestingly, the regions of KHSRP_130‐503_ affected by DNA binding are slightly different for *c‐Myc* and *c‐Kit2*. In particular, the signals of the residues located on the KH3 domain are severely decreased in intensity in the presence of both G4‐forming DNAs. However, only in the presence of *c‐Myc*, several signals corresponding to residues of the KH2 domain experience a large decrease in intensity, while they remain almost unchanged upon the addition of *c‐Kit2*. Conversely, residues located on the KH4 domain experience larger effects in the presence of *c‐Kit2*. Some more modest effects on the KH1 and KH4 domains due to *c‐Myc* DNA were also visible. Moreover, in the presence of both DNA strands, some residues, mostly from the KH3 and KH4 domains, undergo a slight chemical shift perturbation (Figure S15, Supporting Information). The change in chemical shift observed for the residues of the linker between the KH3 and KH4 domains is also intriguing. This could be explained by a conformational change that this loop undergoes upon DNA binding (Figure S16, Supporting Information).

### 2.6 Thermal Properties of KHSRP_130‐503_ in the Presence of *c‐Kit2* G4

Differential scanning calorimetry (DSC) is a valuable tool also for investigating the interaction between a biological macromolecule and a ligand.^[^ ^64^, ^65^ ^]^ It is particularly useful for assessing how ligand binding affects the stability of the macromolecule and allows correlation of the thermodynamics that drive the binding with any conformational changes occurring in the macromolecule due to the interaction. Le Chatelier's principle indicates that if a ligand binds preferentially to the folded form of a biological macromolecule, this will stabilize the folded state, and the unfolding of the macromolecule will become less favorable in the presence of the ligand. In the case of two biological macromolecules, their association to form a complex can result in the stabilization of both components. Examples in the literature of DSC heat capacity profiles show that on heating, protein/DNA complexes can unfold at a somewhat higher temperature than the free proteins and free DNA molecules.^[^ ^65^ ^]^

We have shown above, through CD melting experiments, that the strongest G4‐KHSRP_130‐503_ interactions, specifically with *c‐Kit2* and *c‐Myc* G4s, result in a significant increase in the stability of these DNA structures in the presence of the protein. Since it was not possible to use CD spectroscopy to investigate the thermal properties of the protein in the presence of G4s, due to the simultaneous absorption of DNA at the wavelengths useful for this purpose, we employed DSC.

**Figure 6:**
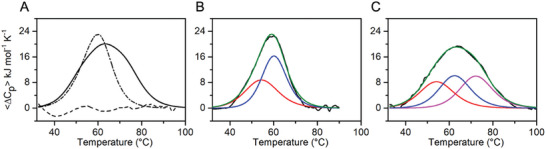
A) Comparison of DSC profiles for KHSRP_130‐503_ (dot‐dashed line), KHSRP_130‐503_/*c‐Kit2* mixture (solid line), and *c‐Kit2* (dashed line). B,C) DSC thermograms for KHSRP_130‐503_ and KHSRP_130‐503_ in complex with *c‐Kit2* G4 (black lines) with corresponding deconvolutions of the excess heat capacity functions. B) Two transitions are observed for free KHSRP_130‐503_ (red and blue lines), and their sum is shown as a green line. *T*_m_ values for the first (red line) and second (blue line) transitions are 54 and 60 °C, respectively. C) Three transitions are observed for complexed KHSRP_130‐503_ (red, blue, and magenta lines) and their sum is shown as a green line. *T*_m_ values for the first (red line), second (blue line), third (magenta line) transitions are ≈54, 62, and 72 °C, respectively. Experiments were performed in 5 mM potassium phosphate buffer (pH 7.0), 20 mM KCl, using a heating rate of 1 °C min^−1^.

The DSC thermograms obtained for the denaturation of KHSRP_130‐503_ in the absence and presence of *c‐Kit2* G4 are shown in **Figure** **6**A. The DSC curves show that protein unfolding proceeds with significant heat absorption, i.e., enthalpy change. As for the free KHSRP_130‐503_, the apparent melting temperature, *T*_m_, corresponding to the maximum of DSC peak (59.0 ± 0.5 °C), coincides with the *T*_m_ obtained by following the temperature dependence of the molar ellipticity at 222 nm (58.0 ± 1.0 °C) (Figure S17, Supporting Information). The latter shows that the helicity of the protein decreases sigmoidally over the range from ≈35 to 75 °C, dropping to a value corresponding to a completely unfolded state, and that the ellipticity also changes at temperatures below 35 °C, suggesting some temperature‐induced conformational change.

The analysis of the DSC profile of KHSRP_130‐503_ showed that the curve is not completely symmetrical and cannot be well described by a two‐state melting process. Deconvolution analysis of the DSC curve revealed that the unfolding of the protein proceeds in two stages (Figure 6B), with the first transition (*T*_m_ ≈ 54 °C) occurring with a smaller enthalpy change than the second (*T*_m_ ≈ 60 °C). Considering the structure of the protein, one could conclude that some domains may be slightly less stable than others. Studies reported in the literature support this hypothesis and suggest that KH1 should be, among the four domains, the significantly less stable one.^[^ ^66^ ^]^

In the presence of *c‐Kit2* G4, it is observed that the complex appears more thermostable than the free protein (apparent *T*_m_ = 63.7 ± 0.5 °C). The calorimetric enthalpy change, obtained by direct integration of the area under the DSC curve, is significantly larger than the value obtained by analyzing the curve of KHSRP_130‐503_ (Δ_cal_ *H* = 531 and 402 kJ mol^−1^, respectively). An important observation is that the thermal transition of free G4 is not detectable at the concentrations used for this experiment (dashed line in Figure 6) and therefore its contribution can be neglected. The DSC curve of the protein/G4 mixture clearly shows a multistate transition, therefore deconvolution of the experimental curve was performed. Such analysis showed that the thermal transition can be modeled by a minimum of three transitions (Figure 6C), the first two of which have *T*_m_ values very close to those of the two transitions observed for the protein alone (*T*_m_ ≈ 54 and 62 °C, respectively), while the third transition occurs at a higher temperature (*T*_m_ ≈ 72 °C). These results suggest that the first two transitions may correspond to protein domains not involved in G4 binding, while the third should be due to the protein domains that interact with DNA in the complex.

### 2.7 Pyridostatin Hinders the Interaction of KHSRP_130‐503_ with *c‐Myc* and *c‐Kit2* G4s

**Figure 7:**
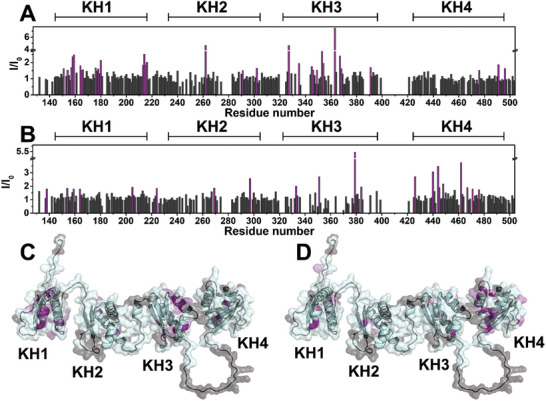
Graphical representation of the per‐residue intensity changes of (A,C) KHSRP_130‐503_/*c‐Myc* and B,D) KHSRP_130‐503_/*c‐Kit2* complexes (50 µm) in the presence of pyridostatin (200 µm for *c‐Myc* and 100 µm *c‐Kit2*) compared to the protein in the presence of *c‐Myc* or *c‐Kit2* alone. The residues exhibiting the highest increases in signal intensities (*c‐Myc*: Val151, Gly154 Gly157, Leu158, Ile159, Gly164, Glu165, Gln166, Cys176, Val178, Ile180, Ser213, Arg214, Gly215, Arg216, Arg262, Lys291, Ile302, Val327, Gly335, Ile345, Lys346, Gln349, Gly353, Val354, Gly363, Glu367, Lys368, Gln391, Ile476, Ile491, Glu496; *cKit2*: His138, Gly154,Ile159, Gly164, Lys205, His224, Leu269, Glu297, Ser333, Asp351, Cys379, Arg384, Glu426, Ile440, Gly444, Glu445, Lys448, Arg462, Asn467, Asn471, Ile476) are colored in violet in the plots (A,B) and on the AlphaFold model of the protein (C,D). Residues that could not be assigned in the NMR spectra are colored in gray on the protein model.

We also evaluated the effect of pyridostatin, a first‐in‐class G4‐binding ligand,^[^ ^67^ ^]^ on the interaction of KHSRP_130‐503_ with *c‐Kit2* and *c‐Myc* G4s. First, NMR titrations of the KHSRP_130‐503_/G4 complexes with the ligand were performed. After the addition of pyridostatin to KHSRP_130‐503_ in complex with G4 DNAs, the protein signals were clearly affected by intensity changes. In detail, a group of the signals that experienced a decrease in intensity after the addition of G4s, partially restored their original intensity upon pyridostatin addition (**Figure** **7**). In the presence of *c‐Myc*, these signals belong to residues located in the KH1 and KH3 domains, whereas in the presence of *c‐Kit2*, the signals influenced by the addition of pyridostatin belong mainly to the KH4 domain. These effects imply that pyridostatin can affect the interaction of KHSRP_130‐503_ with both G4s.

To verify these data using an orthogonal biophysical assay, SPR experiments were again carried out on *c‐Myc* and *c‐Kit2* G4s when complexed to the ligand. SCK experiments were performed on KHSRP_130‐503_–immobilized sensor chip, flowing the G4s in the presence of pyridostatin. Increasing concentrations of the G4/pyridostatin complexes were flowed on the immobilized protein as reported above. A comparison between the resulting sensorgrams and those obtained in the absence of pyridostatin (Figure S18, Supporting Information) reveals that the presence of this G4 binder certainly has a significant impact on the recognition of G4s by the protein.

### 2.8 Biological Validation of KHSRP/G4 Interaction

**Figure 8:**
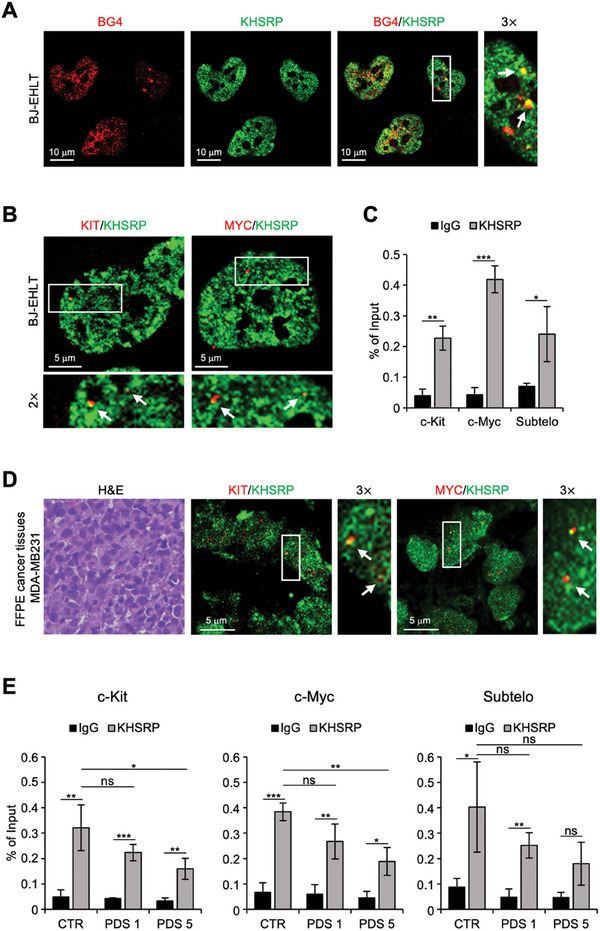
KHSRP binds to G4 structures located within the promoters of *c‐KIT* and *c‐MYC* in cells. A) Representative images of immunofluorescence (IF) analyses performed in human BJ‐EHLT fibroblast using the antibodies against G4 structures (anti‐BG4, red spots) and KHSRP (green spots). Arrows indicate the colocalization (yellow spots). Specific enlargement (2×) is shown. Scale bar: 10 µm. B) Representative images of IF‐combined FISH analysis (IF‐FISH) performed in BJ‐EHLT using specific probes for *c‐Kit* and *c‐Myc* sequences (red spots) and the antibody against KHSRP (green spots). Arrows indicate the colocalization (yellow spots). Specific enlargements (3×) are shown. Scale bar: 5 µm. C) Chromatin Immunoprecipitation (ChIP) assays performed in BJ‐EHLT fibroblasts by immunoprecipitating protein‐DNA complex with the antibody against KHSRP. Rabbit IgG was used as a negative control. KHSRP enrichment at G4 forming sequence of the promoter of c‐KIT and c‐MYC and at subtelomeric regions was evaluated by qPCR Real‐Time analyses. The histogram represents the quantification of KHSRP enrichment, expressed as the percentage of the input. D) Ex vivo analysis on FFPE cancer tissues derived from MDA‐MB‐231 tumor tissue. *Left panel*: representative images of H&E staining; scale bar: 40 µm. *Central and Right panels*: Representative images of IF‐FISH analysis of KHSRP and *c‐Kit* and *c‐Myc* genes performed as in (B). Arrows indicate the colocalization (yellow spots). Specific enlargements (3×) are shown. Scale bar: 5 µm. E) ChIP assays performed in BJ‐EHLT fibroblasts treated with pyridostatin (PDS) for 24 h at the indicate doses and performed as in (C). All representative images of IF and IF‐FISH were acquired by confocal microscopy (magnification 63×). All histograms represent mean values ± S.D. of three independent experiments. *p < 0.05, **p < 0.01, ***p < 0.001.

In the latter part of this study, we aimed to validate the biophysical data by testing the ability of KHSRP to bind G4 structures in cells. For this purpose, BJ‐EHLT cells – a model of transformed human foreskin fibroblasts expressing KHSRP (Figure S19A, Supporting Information) – were subjected to immunofluorescence (IF) experiments to evaluate the co‐localization between the cell‐endogenous KHSRP and the G4 structures. Of note, confocal microscopy analysis evidenced numerous co‐localization spots within the cell nuclei (**Figure** **8**A), indicating that endogenous KHSRP can interact with G4 structures spontaneously originating within the human genome, in line with what was already demonstrated with the truncated form of the protein in vitro. Furthermore, experiments performed by combining immunofluorescence of KHSRP and fluorescence in situ hybridization (FISH) assays – carried out by labeling *c‐KIT* and *c‐MYC* with specific probes – clearly showed that KHSRP localizes in the proximity of these genes (Figure 8B).

Based on these findings, and taking into account the results of biophysical analyses, we focused on gaining deeper insights into the capability of KHSRP to bind the well‐known G4s localized within the promoters of *c‐KIT* and *c‐MYC*. To this aim, we performed a chromatin immunoprecipitation (ChIP) assay to evaluate the amount of KHSRP associated with the G4 regions of interest. For these experiments, BJ‐EHLT cells were first subjected to treatment with a cross‐linking agent (formaldehyde) and, upon stabilization of protein‐DNA interactions, KHSRP was immunoprecipitated and the G4 motifs localized within the promoter of *c‐KIT* and *c‐MYC* were amplified and quantified by real‐time qPCR. Of note, the results evidenced a significant enrichment of KHSRP associated with the G4‐forming sequences of each gene promoter (Figure 8C). For completeness, we also assessed the ability of KHSRP to bind telomeric G4s. Interestingly, the ChIP assays performed with primers designed to pair with sub‐telomeric regions (Table S1, Supporting Information) confirmed that KHSRP also effectively binds telomeric G4s in the cellular environment (Figure 8C), in agreement with our previous findings.^[^ ^46^ ^]^

The same experiments were then extended to HeLa and MCF7 cells, two human cancer cell lines deriving from cervical carcinoma and breast adenocarcinoma, respectively, both expressing KHSRP (Figure S19A, Supporting Information). Interestingly, IF analyses (Figure S19B, Supporting Information), as well as ChIP assays (Figure S19C,D, Supporting Information), fully recapitulated what was previously observed in BJ‐EHLT cells, indicating that the ability of KHSRP to bind the G4 structures located within the promoter regions of *c‐KIT* and *c‐MYC* is a generalizable phenomenon, independent of tumor histotype.

These findings were further confirmed in a xenograft model obtained by the intramuscular injection of MDA‐MB‐231, a breast cancer cell line of human origin, within the leg of immune‐compromised mice. In particular, FISH assays performed on tumor sections highlighted that KHSRP, even in the complexity of an in vivo model, maintains its ability to localize within the sequences of *c‐KIT* and *c‐MYC* (Figure 8D), further underscoring the biological relevance of our findings.

To complete the biological characterization of the interaction between KHSRP and G4s in cells, ChIP assays were performed in BJ‐EHLT cells exposed to increasing concentrations of pyridostatin (1 and 5 µm for 24 h). Interestingly, the G4 ligand was found to impair, in a dose‐dependent manner, the binding of KHSRP to G4 structures (Figure 8E). This indicates that pyridostatin can displace KHSRP from G4s and suggests that they compete for the same binding region on the G4‐forming DNA, consistent with the results of NMR experiments. Altogether, these findings corroborate the direct binding of KHSRP to G4 structures.

## 3 Discussion

G4 structures play a key role in the regulation of diverse cellular processes and therefore are one of the crucial secondary structures in nucleic acids. In particular, DNA G4s have been shown to play significant roles in the complex mechanism of gene expression, in which they participate, along with a network of interacting proteins, in the regulation of transcription. Precise orchestration of G4 formation, stabilization, and resolution is thus imperative for ensuring proper biological functions.

Within this context, the recognition and processing of G4s by nucleic acid‐directed proteins emerge as pivotal events, influencing the activation or deactivation of physiological and pathological pathways. Some proteins act as mediators, detecting G4s and recruiting helicases or other proteins to facilitate the unfolding of noncanonical structures. At the same time, distinct proteins possess the ability to directly recognize and destabilize G4s, while others have the opposite function of stabilizing them. These interactors alter the equilibrium between unfolded G‐rich sequences and folded G4s and make G4 formation very dynamic within living cells.

To date, a wide range of G4‐binding proteins has been discovered. Whilst there are multiple studies describing their identification, for most of them the molecular basis of interactions remains to be understood. Furthermore, while some proteins exhibit a broad nucleic acids‐binding capability, others act in a more selective manner and with different affinity, but virtually all of them have multiple roles. KHSRP has been shown to be among those proteins capable of interacting with multiple RNA and DNA targets, thanks to its modular structure and binding flexibility.

In this investigation, we conducted biophysical and biological studies to gain insights into the G4 DNA recognition by the KHSRP protein. We focused on biologically relevant G4‐forming sequences found in the promoter regions of *c‐MYC*, *c‐KIT*, and *BCL‐2* oncogenes, as well as in human telomeric DNA. Initially, we optimized protein expression and purification, followed by an NMR study of KHSRP_130‐503_, which allowed us to assign backbone residues. Then, we focused on studying G4‐protein interactions in vitro. Using various biophysical methodologies (NMR, SPR, CD melting, and DSC), we found that KHSRP binds to the investigated G4s, albeit with differences in terms of affinity and binding mode. Both protein‐based and DNA‐based 1D ^1^H NMR experiments provided evidence of a better interaction with the parallel‐stranded G4s (*c‐Kit1*, *c‐Kit2*, and *c‐Myc*) compared to hybrid topology G4s (*Bcl‐2* and *mTel_24_*). As for *c‐Kit1*, *c‐Kit2*, and *c‐Myc* G4s, the DNA residues experiencing the most significant perturbations were those belonging to the external G‐tetrads, indicating the involvement of G‐tetrad planes in the protein binding process. This could also explain the reduced effects observed in interactions between the protein and hybrid G4s in which the external G‐tetrads are predominantly occupied by loops, decreasing their susceptibility to interaction.

Interestingly, no unfolding or structural alterations in DNA secondary structures were detected upon binding to KHSRP. Instead, stronger interactions resulted in enhanced thermal stability of the G4 structures induced by the protein, indicating that KHSRP preferentially binds to the folded form of DNA molecules. In this context, it cannot be excluded that KHSRP may serve as a mediator to recruit other proteins, including helicases, either directly or indirectly. For instance, as mentioned in the introduction, KHSRP interacts with hnRNPA1,^[^ ^35^ ^]^ a protein known to resolve G4 structures at telomeres and in the promoters of genes such as *KRAS* and *TRA2B*,^[^ ^40^, ^42^ ^]^ thereby modulating their transcription in cancer cells. Additionally, KHSRP is also part of a multiprotein complex that includes hnRNPF and hnRNPH1, which have recently been shown to interact with RNA G4s.^[^ ^27^ ^]^ While the involvement of these proteins with DNA G4 structures has not yet been demonstrated, this possibility cannot be ruled out.

To gain insights into the thermodynamic and kinetic aspects of binding, the interaction of KHSRP with the five G4s was examined by SPR. These experiments indicated specific DNA/protein interactions and showed that G4 structural variability can significantly influence protein recognition. Indeed, the affinity for parallel‐stranded G4s was found to be at least one order of magnitude higher than that measured for hybrid G4s, in agreement with the NMR data. Despite the different binding affinities, these findings don't exclude the possibility that this protein might have a broad role as G4‐interactor, not being selective for a single G4. The quantitative estimate of on/off rates of binding revealed that the difference in affinity between parallel and hybrid G4s arises mainly from dissociation rates rather than association rates. Given the structural differences between parallel and non‐parallel G4s, the reduced protein affinity for non‐parallel G4s can be attributed to the presence of loop residues above the G‐tetrads. Although these loops do not appear to impede recognition, they may restrict the formation of more extensive or complete interactions. Among the parallel G4s, KHSRP showed the highest affinity for *c‐Kit2*, followed by *c‐Myc*, with kinetic analysis indicating that the difference lies in the association rates.

The 2D NMR data on the interaction of the protein with *c‐Kit2* and *c‐Myc* G4s suggested a major role for the KH3 domain, with varying participation from the other domains depending on the target. This is consistent with a multi‐domain mechanism of KHSRP–G4 recognition, as also observed for KHSRP binding to RNA targets.^[^ ^37^ ^]^ Our calorimetric investigations on the stability of KHSRP in the absence and presence of *c‐Kit2* G4, have shown that the stability of the protein, or rather of some of its domains, changes upon association with the DNA structure, as a consequence of the interaction and the specific involvement of the different domains in the binding to DNA.

Interestingly, the results of our experiments performed in the presence of pyridostatin, a well‐characterized G4 ligand, clearly showed that it can affect G4/protein interactions in vitro and in cells, further corroborating that KHSRP is capable of directly binding to G4 structures. As documented in the literature, G4‐targeting agents are known to modulate gene expression by altering the formation of G4s within gene regulatory elements, such as gene promoters and/or enhancers. However, the impact of pyridostatin on gene expression is quite complex. Indeed, it can cause DNA double‐strand breaks,^[^ ^68^ ^]^ and displace G4‐binding proteins.^[^ ^69^, ^70^ ^]^ Together, these results suggest that pyridostatin may compete with the protein in binding the G4s, potentially influencing other processes resulting from protein‐DNA recognition.

Overall, our results provided valuable insights into the structural and energetic aspects of the KHSRP‐G4 interaction and shed light on the involvement of protein domains in the molecular recognition of these noncanonical DNA structures, as well as on the G4 structural determinants that contribute to the interaction. Additionally, validation of these findings in cancer cells and preclinical in vivo models highlighted their biological relevance, suggesting that KHSRP may play a significant role in cancer formation and progression. This paves the way for further investigations aimed at exploring potential therapeutic developments related to these findings.

## Conflict of Interest

The authors declare no conflict of interest.

## Supporting information



Supporting Information

## Data Availability

The protein resonance assignment has been deposited in the BMRB database under the accession code: 52573. The data that support the findings of this study are available from the corresponding author upon reasonable request.
