# Supplementary material for: Expanding the Functions of KHSRP Protein: Insights into DNA G‐Quadruplex Binding
Source: Adv Sci (Weinh). 2025 Jan 6;12(8):2410086. doi: 10.1002/advs.202410086 (PMC11848572; doi:10.1002/advs.202410086)
Supplement: Supplementary file 1 — Supporting Information [file ADVS-12-2410086-s001.pdf]

ADVANCED SCIENCE
Open Access

Supporting Information

for *Adv. Sci.*, DOI 10.1002/advs.202410086

Expanding the Functions of KHSRP Protein: Insights into DNA G-Quadruplex Binding

*Pasquale Russomanno, Pasquale Zizza, Linda Cerofolini, Federica D'Aria, Sara Iachettini, Serena Di Vito, Annamaria Biroccio, Jussara Amato, Marco Fragai and Bruno Pagano**

Supporting Information

Expanding the Functions of KHSRP Protein: Insights into DNA G-quadruplex Binding

*Pasquale Russomanno, Pasquale Zizza, Linda Cerofolini, Federica D'Aria, Sara Iachettini, Serena Di Vito, Annamaria Biroccio, Jussara Amato, Marco Fragai, and Bruno Pagano**

Table of Contents

**Experimental Section**	S2
**Figure S1**	S10
**Figure S2**	S11
**Figure S3**	S12
**Figure S4**	S13
**Figure S5**	S14
**Figure S6**	S15
**Figure S7**	S16
**Figure S8**	S17
**Figure S9**	S18
**Figure S10**	S19
**Figure S11**	S20
**Figure S12**	S21
**Figure S13**	S21
**Figure S14**	S22
**Figure S15**	S23
**Figure S16**	S24
**Figure S17**	S25
**Figure S18**	S25
**Figure S19**	S26
**Table S1**	S27
**References**	S28

Experimental Section

Expression and purification of the full-length human KHSRP protein

The plasmid encoding the full-length KHSRP protein ($KHSRP_{fl}$, residues 1-711) (Figure S1) was cloned into the pGEX-6P-1 vector (Twin Helix srl, Milan, Italy) containing a GST tag between the BamHI and NotI sites and overexpressed in *Escherichia coli* BL21(DE3) gold strain cells (Figure S2). pGEX-6P-1 transformed cells were poured into 1 L of LB medium supplied with ampicillin. A drop of antifoam was added, and the culture was let shake at 310 K and 180 rpm until optical density at 600 nm (OD_{600}) reached 0.60, then cooled to 291 K. Protein overexpression (OD_{600} = 1.6-1.8) was induced by the addition of 0.5 mM IPTG overnight, then cells were harvested by centrifugation at 6500 rpm for 45 min at 277 K. The pellet was resuspended in PBS buffer supplied with the protease inhibitor (Roche), 10% (v/v) glycerol, and 1 mM DTT. The suspension was sonicated on ice for 45 cycles, alternating 10 s of pulse and 30 s of resting, then ultracentrifuged at 18000 rpm at 277 K for 45 min. The supernatant was cyclically loaded onto a GST-column for an initial purification based on the GST-affinity chromatography. Several elution steps using different buffers were performed to obtain the protein free from the bacterial DNA. After elution with the 50 mM Tris-HCl buffer, pH 7.4, containing 500 mM NaCl, and 10 mM GSH, the GST tag was removed by PreScission protease treatment, and the cleaved protein was purified by size exclusion chromatography on a Hi-Load 16/600 Superdex 75pg column (GE Healthcare), previously equilibrated with the 50 mM Tris-HCl buffer, pH 7.4, containing 500 mM NaCl. Elution was performed at 1 mL/min and fractions containing $KHSRP_{fl}$ were identified by SDS-PAGE using Coomassie staining and combined. The folding of the protein was checked by nuclear magnetic resonance (NMR). The solution of protein was stored at 277 K.

Expression and purification of the human $KHSRP_{130-503}$ protein

The plasmid encoding $KHSRP_{130-503}$ (residues 130-503) was cloned into the pET-28a(+) vector (Twist Bioscience, San Francisco, CA) containing a His-tag between the NdeI and XhoI sites and overexpressed in *Escherichia coli* BL21(DE3) gold strain cells (Figure S2). pET-28a(+) transformed cells were poured into 1 L of LB medium supplied with kanamycin for the expression of the protein in natural isotopic abundance, or M9 minimal medium supplied with kanamycin, 2.0 mM $MgSO_4$, 0.2 mM $CaCl_2$, 3.0 g of glucose, 1 mg of thiamine, 1 mg of biotin, and 1.2 g of ^{15}N-ammonium sulfate for the expression of ^{15}N isotopically enriched protein. In both media, a drop of antifoam was added, and the culture was let shake at 310 K and 180 rpm until reaching an OD_{600} value of 0.6, then cooled to 291 K. Protein overexpression (unlabeled and ^{15}N-labeled forms) was induced with 0.5 mM IPTG and further shaking the cell culture overnight at 180 rpm and 291 K. Cells were harvested by

centrifugation at 6500 rpm for 45 min at 277 K. The purification method was the same for both unlabeled and ^{15}N-labeled protein: the pellet was resuspended in 50 mM Tris-HCl buffer, pH 7.5, containing 500 mM NaCl, 5 mM imidazole, and supplied with the protease inhibitor, 10% (v/v) glycerol, and 1 mM DTT. The suspension was sonicated for 45 cycles alternating 10 s of pulse and 30 s of resting, then ultracentrifuged at 18000 rpm at 277 K for 45 min. The supernatant was cyclically loaded onto a Ni^{2+}-column (HiTrap HP column, GE Healthcare) for the purification, and the protein eluted with a linear gradient buffer from 0 to 100% of 50 mM Tris-HCl buffer, pH 7.5, containing 500 mM NaCl, and 500 mM imidazole in 40 min. The collected fractions of protein were merged, then buffer was exchanged with 20 mM Tris–HCl buffer, pH 7.5, containing 200 mM NaCl, and 0.5 mM EDTA by using an HiPrep™ 26/10 desalting column (GE Healthcare). Finally, a stock solution of TEV protease was added to cleave the His-tag and the mixture incubated overnight at 277 K. The solution was concentrated and purified by size exclusion chromatography on a Hi-Load 16/600 Superdex 75pg column (GE Healthcare), previously equilibrated with 5 mM KH_2PO_4/K_2HPO_4 buffer, pH 7.0, containing 20 mM KCl. Elution was performed at 1 mL/min, the fractions containing $KHSRP_{130\text{-}503}$ were identified by SDS-PAGE using Coomassie staining and combined. The folding of the protein (unlabeled and ^{15}N-labeled forms) was checked by NMR. Finally, the solution of protein was aliquoted, lyophilized and stored at 253 K until use.

Preparation of DNA samples

DNA oligonucleotides were obtained by chemical synthesis at 1 μmol scale on an ABI 394 DNA/RNA synthesizer (Applied Biosystem), using the standard β-cyanoethylphosphoramidite solid phase chemistry, followed by purification as described elsewhere.[1] The following oligonucleotides were synthesized: d(AGGGAGGGCGCTGGGAGGAGGG) (*c-Kit1*) and d(CGGGCGGGCGCTAGGGAGGGT) (*c-Kit2*) from the *c-KIT* oncogene promoter, d(TGAGGGTGGGTAGGGTGGGTAA) (*c-Myc*) from the *c-MYC* promoter sequence, d(GGGCGCGGGAGGAATTGGGCGGG) (*Bcl-2*) from the *BCL-2* promoter sequence, and the modified human telomeric sequence d(TTGGGTTAGGGTTAGGGTTAGGGA) ($mTel_{24}$).[2–6] The isolated oligomers were proved to be >98% pure by NMR. The concentration of oligonucleotides was determined by UV adsorption measurements at 90 °C using appropriate molar extinction coefficient values ε (λ = 260 nm), calculated by the nearest-neighbor model.[7] All G4s were prepared in 5 mM KH_2PO_4/K_2HPO_4 buffer containing 20 mM KCl (pH 7.0). Samples were then heated at 90 °C for 5 min, gradually cooled to room temperature overnight, and finally incubated at 4 °C for 24 h, before data acquisition.

Circular dichroism (CD) spectroscopy experiments

CD experiments were performed using a Jasco J-815 spectropolarimeter (Jasco Inc.) equipped with a PTC-423S/15 Peltier temperature controller. Spectra were recorded at 20 °C in a 1 mm path-length quartz cuvette in the wavelength range of 200–340 nm, with a scan rate of 20 nm/min, response time of 1 s, and 1 nm bandwidth. Spectra were averaged over three scans. Buffer baseline was subtracted from each spectrum. The DNA concentration used for each experiment was 5 μM in 5 mM KH_2PO_4/K_2HPO_4 buffer containing 20 mM KCl, pH 7.0. Measurements were carried out in the absence or presence of 5 μM $KHSRP_{130\text{-}503}$. DNA/protein mixtures were allowed to equilibrate for 15 min before data acquisition. The CD spectrum of the protein alone (5 μM) was also recorded. CD melting experiments of G4s in the absence or presence of equivalent amounts of $KHSRP_{130\text{-}503}$ were performed at 1 °C/min heating rate by following the CD signal at the wavelength of maximum intensity in the spectral region where only the G4 chromophores absorb (263 nm for *c-Kit1*, *c-Kit2*, and *c-Myc*; 266 nm for *Bcl-2*; 290 nm for *mTel*$_{24}$). Data analysis was performed using OriginPro 2021 software (OriginLab Corp., MA, USA).

Nuclear magnetic resonance (NMR) spectroscopy experiments

1D ^{1}H-NMR experiments. The 1D ^{1}H-NMR spectra of $KHSRP_{fl}$ and $KHSRP_{130\text{-}503}$, and the whole set of 1D ^{1}H macromolecule-based NMR experiments were acquired on a Bruker AVANCE NEO NMR spectrometer, operating at 600 MHz (^{1}H Larmor frequency), equipped with a SampleJet autosampler and a 5 mm QCI H-P/C/N-D-5-Z CryoProbe, optimized for ^{1}H sensitivity. All spectra were acquired at 298 K, using 512 scans per spectrum with a recovery delay of 1.5 s. The water signal was suppressed using the excitation sculpting with gradients.[8] Spectra were phase adjusted, baseline corrected, and calibrated with respect to the water frequency. Processing and analysis were performed using the software package Bruker TOPSPIN 4.0.7. Experiments for assessing the folding of $KHSRP_{fl}$ and $KHSRP_{130\text{-}503}$ were carried out using 20 μM of each protein in the corresponding 9:1 H_2O/D_2O buffer ($KHSRP_{fl}$: 50 mM Tris-HCl buffer, pH 7.4, containing 500 mM NaCl; $KHSRP_{130\text{-}503}$: 5 mM KH_2PO_4/K_2HPO_4 buffer, pH 7.0, containing 20 mM KCl). 1D ^{1}H macromolecule-based NMR screening experiments were carried out using 20 μM $KHSRP_{130\text{-}503}$ or G4 DNA (in protein- or DNA-based assays, respectively) in 5 mM KH_2PO_4/K_2HPO_4 buffer, pH 7.0, containing 20 mM KCl and 10% D_2O. Spectra were recorded in the absence and presence of stoichiometric amounts of the putative interacting partner (20 μM DNA or protein, in protein- or DNA-based assays, respectively).

Protein backbone assignment. Solution NMR experiments for backbone resonance assignment [3D HNCA, HNCACB, CBCA(CO)NH, HNCO, HN(CA)CO][9–11] were recorded on a [U-^{13}C, ^{15}N] sample of $KHSRP_{130\text{-}503}$ (at the concentration of 200 μM) in a buffer solution of 5 mM

KH_2PO_4/K_2HPO_4 (pH 7.0), containing 20 mM KCl, 0.1% NaN_3, 1 mM protease inhibitors (Roche) and 10% D_2O. For 3D CBCA(CO)NH and HN(CA)CO, non-uniform random sampling at 48% and 56%, respectively, was used; compressed-sensing reconstruction was then applied.[12] All NMR spectra were recorded at 298 K on a Bruker AVANCE NEO NMR spectrometer, operating at 900 MHz, ^{1}H Larmor frequency (21.1 T), equipped with a 5 mm TCI triple resonance cryo-probe. Two-dimensional carbon-detected (Hα)CON, (H^N)CON, (H)CACO, (H)CBCACO NMR spectra were acquired on a Bruker AVANCE NEO 1200 MHz spectrometer, ^{1}H Larmor frequency (28.2 T) equipped with a triple-resonance 5 mm TXO cryo-probe optimized for ^{13}C-direct detection, to help in the assignment of some proline signals (Figure S6).[13,14] All the spectra were processed with the Bruker TopSpin 4.0.6 software and analyzed with the program CARA.[15] Secondary structure prediction was performed with TALOS+,[16] by using the chemical shifts of HN, N, C', Cα, and Cβ atoms as input data.

Interaction of the KHSRP with c-Myc and c-Kit2 DNA G4s evaluated through 2D ^{1}H-^{15}N HSQC NMR experiments. The interactions of $KHSRP_{130-503}$ with *c-Myc* and *c-Kit2* were also investigated by solution NMR, acquiring 2D ^{1}H-^{15}N HSQC NMR spectra. During the NMR titrations, increasing aliquots of the DNA G4s (to reach the concentrations of 25, 50, and 100 μM for *c-Myc*, and 6.25, 12.5, 25, 50, and 100 μM for *c-Kit2*) were added to the solution of free [U-^{15}N] KHSRP (at a protein concentration of 50 μM in 5 mM KH_2PO_4/K_2HPO_4 buffer, pH 7.0, containing 20 mM KCl and 10% D_2O). 2D ^{1}H-^{15}N HSQC NMR spectra were recorded after each DNA addition on a Bruker AVANCE NEO NMR spectrometer, operating at 900 MHz, ^{1}H Larmor frequency (21.1 T), equipped with a 5 mm TCI triple resonance cryo-probe. In addition, NMR titrations of the $KHSRP_{130-503}$/*c-Myc* and $KHSRP_{130-503}$/*c-Kit2* complexes with the well-known G4 ligand pyridostatin were performed. During the NMR titrations, increasing aliquots of pyridostatin (to reach the concentrations of 25, 50, 100, 200, 400 μM for the $KHSRP_{130-503}$/*c-Myc* complex, and 50, 100 μM for the $KHSRP_{130-503}$/*c-Kit2* complex) were added to the solution of [U-^{15}N] KHSRP (at protein concentration of 50 μM in 5 mM potassium phosphate, pH 7.0, containing 20 mM KCl and 10% D_2O) in the presence of 50 μM of *c-Myc* or *c-Kit2*. 2D ^{1}H-^{15}N HSQC NMR spectra were recorded after each addition on a Bruker AVANCE NEO NMR spectrometer, operating at 900 MHz, ^{1}H Larmor frequency (21.1 T), equipped with a 5 mm TCI triple resonance cryo-probe. The 2D ^{1}H-^{15}N HSQC spectra for the interaction study were acquired at 298 K, using 40 scans per spectrum, with a recovery delay of 1.2 s, and acquisition time of 80 and 24 ms on the ^{1}H and ^{15}N dimensions, respectively. All the spectra were processed with the Bruker TopSpin 4.0.6 software and analyzed with the program CARA.[15]

Surface plasmon resonance (SPR) experiments

SPR experiments were performed at 25 °C on a Biacore X100 instrument (Cytiva, USA). The $KHSRP_{130\text{-}503}$ protein was immobilized on a standard CM5 sensor chip using amine-coupling chemistry and HBS-EP as running buffer (HEPES 10 mM, NaCl 150 mM, EDTA 3 mM, 0.005% Surfactant P20, pH 7.4). A 1:1 mixture of 0.1 M NHS (*N*-hydroxysuccinimide) and 0.1 M EDC (3-(*N*,*N*-dimethylamino)propyl-*N*-ethylcarbodiimide) at a flow rate of 10 µL/min was used to activate the surfaces of flow cells. Afterward, the protein (10 µg/mL in 10 mM sodium acetate, pH 4.5), was immobilized at a density of ~1000 RU on the sample flow cell, leaving the reference cell as blank. The unreacted groups were blocked by injection of 1.0 M ethanolamine at 10 µL/min over the chip surface. Kinetic binding data were collected by using the single-cycle kinetics approach.[17] G4s or G4/pyridostatin (1:2) complexes were injected at various concentrations (from 1.25 to 20 µM), using 5 mM KH_2PO_4/K_2HPO_4 buffer (pH 7.0) containing 20 mM KCl as running solution. Injections were performed at a flow rate of 30 µL/min with association and dissociation times of 30 and 600 s, respectively. No regeneration after each sample was required. Data were fitted to a 1:1 kinetic interaction model using the global data analysis option available within the Biacore Evaluation software (Cytiva, USA) provided with the instrument. Spikes were not removed since, as already shown, they do not affect the results.[18,19] The data are the average of three independent experiments with standard deviations.

Differential scanning calorimetry (DSC) experiments

DSC experiments were carried out using a Nano-DSC (TA Instruments, New Castle, DE, USA). The experiments were performed at 20 µM $KHSRP_{130\text{-}503}$ concentration in 5 mM KH_2PO_4/K_2HPO_4 buffer, pH 7.0, with 20 mM KCl, in the absence and presence of *c-Kit2* G4 (20 µM). Scans were performed at 1 °C/min in the 20−100 °C temperature range. A second scan of the samples showed no reversibility for the thermal unfolding of the protein. A buffer−buffer scan, under the same experimental conditions, was subtracted from the corresponding buffer−sample scans, and the baseline was drawn for each scan. The apparent melting temperature (T_m) values were determined from the maximum of each thermogram peak. The model-independent transition enthalpies ($\Delta_{cal}H$) were obtained by integrating the area under the excess heat capacity versus temperature curves. The analysis of the DSC curves was accomplished as already described.[20]

Cell cultures and treatment

The human cancer cell lines deriving from a cervical carcinoma (HeLa) and a breast adenocarcinoma (MCF-7) were purchased from the American Type Culture Collection (ATCC). The human BJ-EHLT

fibroblasts were obtained by infecting BJ cells, purchased from ATCC, first with a retrovirus carrying hTERT and successively with SV40 early region.[21] All mentioned cell lines were cultured in Dulbecco's modified Eagle's medium (DMEM, EuroClone, Milan, Italy; ECM0728L), supplemented with L-glutamine, penicillin-streptomycin and 10% fetal bovine serum (FBS, Thermo Fisher Scientific - Gibco, Waltham, MA, USA), in a CO_2-humidified incubator at 37 °C. Regarding the experiments performed in the presence of pyridostatin (PDS, Selleckchem, Houston, TX, USA), the cells were treated for 24 h at a final drug concentration of 1 and 5 µM.

Immunofluorescence (IF)

Cells were grown on glass coverslips, fixed in 4% formaldehyde in 1× phosphate-buffered saline (1× PBS) for 15 min at room temperature (RT) and permeabilized with 0.25% Triton X-100 (Sigma-Aldrich, St. Louis, MO, USA, 93443) for 5 min at RT. Cells were blocked for 1 h in 3% BSA, 0.1% Tween-20 (1× PBS) and washed twice with PBS. For immune labelling, cells were incubated with mouse anti-DNA/RNA G4 structures (BG4; Absolute Antibody, Oxford, UK, #Ab00174-1.1, 1:2000) and rabbit anti-KHSRP (Novus Biologicals, Easter Ave Centennial, CO, USA, NBP1-18910, 1:200) antibodies in 3% BSA, 0.1% Tween-20 (1× PBS) for 1 h at RT. Next, cells were washed with 0.3% BSA, 0.1% Tween-20 (1× PBS) and incubated for 1 h with anti-mouse Alexa Fluor 555 Conjugate and anti-rabbit Alexa Fluor 488 Conjugate (Cell Signaling, Danvers, Massachusetts, USA, 1:500) antibodies. Finally, nuclei were stained with Duolink In Situ Mounting Medium with DAPI (Sigma Aldrich, St. Louis, MO, USA, DUO82040). Fluorescence signals were acquired by confocal laser scanning microscopy using Zeiss Laser Scanning Microscope 510 Meta (63× magnification) (Zeiss, Jena, Germany).

IF-combined DNA fluorescence *in situ* hybridization (FISH)

Cells were subjected to consecutive fixations with 4% formaldehyde, then were dehydrated with increasing alcohols and allowed to dry at RT. Samples, to which MYC/KIT (MYC-20-OR, KIT-20-OR, Empire Genomics, Buffalo, New York, USA) probes solution was added, were denatured for 5 min at 80 °C and incubated overnight (ON) in a humidified chamber. After the incubation period, slides were first placed in Wash Buffer 1 (0.4× SSC, 0.1% Igepal) at 73 °C for 2 min, then washed with Wash Buffer 2 (2× SSC, 0.1% Igepal) at RT. Immediately afterward, the classical immunofluorescence protocol described above was performed, using the primary rabbit anti-KHSRP antibody (Novus Biologicals, NBP1-18910, 1:200). The final phase involved the denaturation of the samples with increasing concentrations of alcohols and the subsequent mounting of the slides with Duolink In Situ Mounting Medium with DAPI (Sigma Aldrich, DUO82040). Colocalizations of the

probes with KHSRP were analyzed with the Zeiss Laser Scanning Microscope 510 Meta (63× magnification) confocal microscope.

Tissue processing

The sections of breast cancer tissue were obtained from tumor samples already available in our laboratory. Xenografts were originated as reported by Dinami *et al.*,[22] by injecting 4 $\times 10^6$ human breast cancer cell lines (MDA-MB-231) in CB17-SCID (CB17/Icr-Prkdcscid/IcrIcoCrl, 6 weeks old) female mice (Charles River Laboratories, Calco, Italy). Briefly, the collected tissue samples were fixed in 10% neutral buffered formalin for at least 24 h. The specimens were then embedded in paraffin blocks, allowing for sectioning into thin slices (approximately 4-5 μm thick) using a microtome. These sections were carefully transferred onto glass slides and dried at RT to facilitate adherence.

IF-combined FISH analysis. The 4 μm tissue sections were immersed in xylene to dissolve the paraffin, then in a decreasing series of 100%, 85%, and 70% ethanol. The slides were incubated for 15 min at 96-98 °C in a 0.01 M sodium citrate solution and then in a pepsin solution for approximately 30 min. Following a 5 min wash in distilled water at RT and a 1 min rinse in 2× SSC, sections were dehydrated with 70%, 85%, and 100% alcohols and allowed to air dry. The protocol continued as previously described for cell cultures.

Hematoxylin and Eosin (H&E) staining. Sections were deparaffinized by immersion in xylene, followed by rehydration through graded alcohol solutions (100%, 95%, and 70%) and finally rinsing in distilled water. Samples were then stained with hematoxylin solution (Sigma-Aldrich, H9627), a basic dye that binds to nucleic acids and stains cell nuclei blue-purple. After staining time, the excess hematoxylin was removed by rinsing the sections in running water. Following hematoxylin staining, sections were counterstained with eosin (Sigma-Aldrich, HT110216), an acidic dye that imparts a pink color to cytoplasmic components. After eosin staining, the sections were dehydrated through a series of alcohol solutions (70%, 95%, and 100% ethanol) to remove excess water, followed by clearing in xylene to make the tissues transparent. Finally, the sections were coverslipped using a mounting medium to preserve the staining and prepare them for microscopic examination. The representative histological image was acquired with Aperio Slides Scanner CS2 (Leica Biosystems, Nussloch, DE).

Western blot

Western blot analyses were performed as previously reported.[23] Briefly, total protein extracts were obtained from the lysis of cells with a buffer containing 50 mM Tris-HCl (pH 7.5), 5 mM EDTA,

250 mM NaCl, and 0.1% Triton, and completed with protease and phosphatase inhibitors (A32953 and 88667, respectively; Thermo Fisher Scientific). After the SDS-polyacrylamide gel electrophoresis and nitrocellulose filter transfer, the samples were subjected to immunoblot assay. The following primary antibodies were used: rabbit anti-KHSRP (Novus Biologicals, NBP1-18910, 1:1000) and mouse anti-β-actin (Sigma Aldrich, A2228, 1:20000). The following secondary antibodies were used: Goat anti-rabbit and anti-mouse immunoglobulin G (IgG)-horseradish peroxidase-conjugated antibodies (Biorad, 1706515 and 1706516 respectively).

Chromatin immunoprecipitation (ChIP) assay

Cells were seeded and, after 24 h, fixed with 1% of formaldehyde, collected, lysed with SDS lysis buffer (0.1 M NaCl, 1 mM EDTA, 0.5 mM EGTA and 50 mM Tris-HCl pH 8.0) and sonicated 5 times for 20 s setting a high level by using the biorupter standard (Diagenode Inc., NXT-Dx Belgium) to generate fragments averaging from 0.5 to 1 kb. For each immunoprecipitation condition, 100 μg of chromatin and 4 μg of the specific antibodies were diluted in a buffer containing 0.1% SDS, 1% Triton, 1.2 mM EDTA, 16.7 mM Tris-HCl pH 8.0 and 140 mM NaCl. The following antibodies were used: rabbit anti-KHSRP (Novus Biologicals, NBP1-18910) and IgG Rabbit (Bethyl Laboratories, Montgomery, TX, USA) as negative control. After the elution from immuno-complexes and the reverse of cross-linking, the DNA was precipitated and analyzed by qPCR method using SYBR™ Green PCR Master Mix (Applied Biosystems, Foster City, CA, USA; 4309155) in the QuantStudio 6 Flex Detection system (Thermo Fisher Scientific). The list of primers is reported in Table S1. ChIP analysis was performed using the percent Input method.

ATG AGC GAC TAT AGC ACC GGT GGC CCG CCG CCG GGT CCG CCG CCG CCG GCG GGC GGT GGT
1 M S D Y S T G G P P P G P P P P A G G G 20
GGC GGC GCG GGT GGT GCG GGC GGC GGT CCG CCG CCG GGC CCG CCG GGC GCG GGT GAC CGT
21 G G A G G A G G G P P P G P P G A G D R 40
GGT GGT GGC GGT CCG GGT GGT GGT GGC CCG GGC GGC GGT AGC GCG GGT GGC CCG AGC CAA
41 G G G G P G G G G P G G G S A G G P S Q 60
CCG CCG GGT GGT GGT GGT CCG GGT ATC CGT AAG GAT GCG TTT GCG GAT GCG GTG CAG CGT
61 P P G G G G P G I R K D A F A D A V Q R 80
GCG CGT CAG ATT GCG GCG AAA ATT GGC GGT GAC GCG GCG ACC ACC GTG AAC AAC AGC ACC
81 A R Q I A A K I G G D A A T T V N N S T 10
CCG GAT TTT GGT TTC GGT GGC CAA AAA CGT CAG CTG GAA GAT GGT GAC CAG CCG GAA AGC
101 P D F G F G G Q K R Q L E D G D Q P E S 120
AAG AAA CTG GCG AGC CAG GGC GAT AGC ATC AGC AGC CAG CTG GGC CCG ATT CAC CCG CCG
121 K K L A S Q G D S I S S Q L G P I H P P 140
CCG CGT ACC AGC ATG ACC GAG GAA TAC CGT GTT CCG GAT GGT ATG GTT GGC CTG ATC ATC
141 P R T S M T E E Y R V P D G M V G L I I 160
GGC CGT GGC GGT GAA CAG ATC AAC AAG ATT CAA CAA GAC AGC GGT TGC AAG GTG CAA ATT
161 G R G G E Q I N K I Q Q D S G C K V Q I 180
AGC CCG GAC AGC GGT GGC CTG CCG GAA CGT AGC GTG AGC CTG ACC GGT GCG CCG GAA AGC
181 S P D S G G L P E R S V S L T G A P E S 200
GTT CAA AAG GCG AAA ATG ATG CTG GAC GAT ATC GTT AGC CGT GGC CGT GGT GGT CCG CCG
201 V Q K A K M M L D D I V S R G R G G P P 220
GGC CAA TTC CAC GAC AAC GCG AAC GGT GGC CAA AAC GGC ACC GTT CAG GAG ATC ATG ATT
221 G Q F H D N A N G G Q N G T V Q E I M I 240
CCG GCG GGT AAA GCG GGT CTG GTT ATC GGC AAG GGT GGC GAG ACC ATT AAA CAG CTG CAA
241 P A G K A G L V I G K G G E T I K Q L Q 260
GAG CGT GCG GGC GTT AAG ATG ATT CTG ATC CAG GAC GGC AGC CAG AAC ACC AAC GTG GAT
261 E R A G V K M I L I Q D G S Q N T N V D 280
AAG CCG CTG CGT ATT ATC GGT GAT CCG TAT AAG GTG CAG CAG GCG TGC GAG ATG GTG ATG
281 K P L R I I G D P Y K V Q Q A C E M V M 300
GAC ATT CTG CGT GAG CGT GAT CAA GGC GGT TTC GGT GAT CGT AAC GAG TAT GGT AGC CGT
301 D I L R E R D Q G G F G D R N E Y G S R 320
ATT GGT GGC GGT ATT GAC GTT CCG GTG CCG CGT CAC AGC GTT GGT GTG GTT ATT GGT CGT
321 I G G G I D V P V P R H S V G V V I G R 340
AGC GGT GAA ATG ATT AAA AAG ATC CAG AAC GAC GCG GGC GTT CGT ATC CAG TTC AAG CAA
341 S G E M I K K I Q N D A G V R I Q F K Q 360
GAT GAC GGC ACC GGT CCG GAA AAG ATC GCG CAC ATT ATG GGT CCG CCG GAT CGT TGC GAG
361 D D G T G P E K I A H I M G P P D R C E 380
CAC GCG GCG CGT ATT ATC AAC GAC CTG CTG CAA AGC CTG CGT AGC GGC CCG CCG GGT CCG
381 H A A R I I N D L L Q S L R S G P P G P 400
CCG GGC GGT CCG GGC ATG CCG CCG GGC GGC CGT GGT CGT GGT CGT GGT CAG GGT AAC TGG
401 P G G P G M P P G G R G R G R G Q G N W 420
GGC CCG CCG GGC GGT GAA ATG ACC TTC AGC ATC CCG ACC CAC AAA TGC GGT CTG GTG ATC
421 G P P G G E M T F S I P T H K C G L V I 440
GGT CGT GGC GGC GAG AAC GTT AAG GCG ATT AAC CAG CAG ACC GGT GCG TTT GTG GAG ATC
441 G R G G E N V K A I N Q Q T G A F V E I 460
AGC CGT CAA CTG CCG CCG AAC GGC GAC CCG AAC TTC AAG CTG TTC ATC ATC CGT GGC AGC
461 S R Q L P P N G D P N F K L F I I R G S 480
CCG CAA CAG ATC GAC CAC GCG AAG CAG CTG ATC GAA GAG AAA ATC GAG GGT CCG CTG TGC
481 P Q Q I D H A K Q L I E E K I E G P L C 500
CCG GTG GGT CCG GGC CCG GGT GGC CCG GGT CCG GCG GGT CCG ATG GGC CCG TTC AAC CCG
501 P V G P G P G G P G P A G P M G P F N P 520
GGT CCG TTT AAC CAG GGT CCG CCG GGT GCG CCG CCG CAC GCG GGC GGC CCG CCG CCG CAC
521 G P F N Q G P P G A P P H A G G P P P H 540
CAA TAC CCG CCG CAA GGT TGG GGC AAC ACC TAC CCG CAG TGG CAG CCG CCG GCG CCG CAC
541 Q Y P P Q G W G N T Y P Q W Q P P A P H 560
GAT CCG AGC AAG GCG GCG GCG GCG GCG GCG GAC CCG AAC GCG GCG TGG GCG GCG TAT TAC
561 D P S K A A A A A A D P N A A W A A Y Y 580
AGC CAC TAC TAC CAA CAG CCG CCG GGC CCG GTG CCG GGC CCG GCG CCG GCG CCG GCG GCG
581 S H Y Y Q Q P P G P V P G P A P A P A A 600
CCG CCG GCG CAG GGC GAA CCG CCG CAG CCG CCG CCG ACC GGT CAA AGC GAC TAC ACC AAA
601 P P A Q G E P P Q P P P T G Q S D Y T K 620
GCG TGG GAA GAG TAT TAC AAA AAG ATC GGT CAG CAA CCG CAG CAA CCG GGC GCG CCG CCG
621 A W E E Y Y K K I G Q Q P Q Q P G A P P 640
CAA CAG GAC TAT ACC AAG GCG TGG GAG GAA TAT TAC AAG AAA CAG GCG CAG GTT GCG ACC
641 Q Q D Y T K A W E E Y Y K K Q A Q V A T 660
GGT GGC GGC CCG GGT GCG CCG CCG GGT AGC CAG CCG GAC TAT AGC GCG GCG TGG GCG GAA
661 G G G P G A P P G S Q P D Y S A A W A E 680
TAT TAC CGT CAA CAG GCG GCG TAC TAT GGC CAA ACC CCG GGT CCG GGT GGC CCG CAA CCG
681 Y Y R Q Q A A Y Y G Q T P G P G G P Q P 700
CCG CCG ACC CAA CAG GGC CAA CAA CAA GCG CAG TAA
701 P P T Q Q G Q Q Q A Q - 711

Figure S1. Gene and amino acids sequences of full length KHSRP protein.

(A) MSPILGYWKIKGLVQPTRLLLEYLEEKYEEHLYERDEGDKWRNKKFELGLEFPNLPYYIDGDVKLTQSMAIIRYIADKHNMLG
GCPKERAEISMLEGAVLDIRYGVSRIAYSKDFETLKVDFLSKLPEMLKMFEDRLCHKTYLNGDHVTHPDFMLYDALDVVLYMD
PMCLDAFPKLVCFKKRIEAIPQIDKYLKSSKYIAWPLQGWQATFGGGDHPPKSD**LEVLFQGPLG**MSDYSTGGPPPGPPPPAGG
GGGAGGAGGGPPPGPPGAGDRGGGGPGGGGPGGGSAGGPSQPPGGGGPGIRKDAFADAVQRARQIAAKIGGDAATTVNNSTPD
FGFGGQKRQLEDGDQPESKKLASQGDSISSQLGPIHPPPRTSMTEEYRVPDGMVGLIIGRGGEQINKIQQDSGCKVQISPDSG
GLPERSVSLTGAPESVQKAKMMLDDIVSRGRGGPPGQFHDNANGGQNGTVQEIMIPAGKAGLVIGKGGETIKQLQERAGVKMI
LIQDGSQNTNVDKPLRIIGDPYKVQQACEMVMDILRERDQGGFGDRNEYGSRIGGGIDVPVPRHSVGVVIGRSGEMIKKIQND
AGVRIQFKQDDGTGPEKIAHIMGPPDRCEHAARIINDLLQSLRSGPPGPPGGPGMPPGGRGRGRGQGNWGPPGGEMTFSIPTH
KCGLVIGRGGENVKAINQQTGAFVEISRQLPPNGDPNFKLFIIRGSPQQIDHAKQLIEEKIEGPLCPVGPGPGGPGPAGPMGP
FNPGPFNQGPPGAPPHAGGPPPHQYPPQGWGNTYPQWQPPAPHDPSKAAAAAADPNAAWAAYYSHYYQQPPGPVPGPAPAPAA
PPAQGEPPQPPPTGQSDYTKAWEEYYKKIGQQPQQPGAPPQQDYTKAWEEYYKKQAQVATGGGPGAPPGSQPDYSAAWAEYYR
QQAAYYGQTPGPGGPQPPPTQQGQQQAQ

(B) MGSSHHHHHHSSG**LVPRGS**HMGTMTSLYKKAGSAAAVLE**ENLYFQG**GMISSQLGPIHPPPRTSMTEEYRVPDGMVGLIIGRGG
EQINKIQQDSGCKVQISPDSGGLPERSVSLTGAPESVQKAKMMLDDIVSRGRGGPPGQFHDNANGGQNGTVQEIMIPAGKAGL
VIGKGGETIKQLQERAGVKMILIQDGSQNTNVDKPLRIIGDPYKVQQACEMVMDILRERDQGGFGDRNEYGSRIGGGIDVPVP
RHSVGVVIGRSGEMIKKIQNDAGVRIQFKQDDGTGPEKIAHIMGPPDRCEHAARIINDLLQSLRSGPPGPPGGPGMPPGGRGR
GRGQGNWGPPGGEMTFSIPTHKCGLVIGRGGENVKAINQQTGAFVEISRQLPPNGDPNFKLFIIRGSPQQIDHAKQLIEEKIE
GPLCPVG

Figure S2. KHSRP constructs. (A) GST-fused $KHSRP_{fl}$ sequence. Underlined sequence corresponds to GST. Bold part indicates the PreScission protease cleavage site. (B) His-tag-fused $KHSRP_{130-503}$ sequence. Underlined sequence corresponds to His-tag. Bold and underlined part indicates the thrombin cleavage site, whereas the bold sequence indicates the TEV cleavage site.

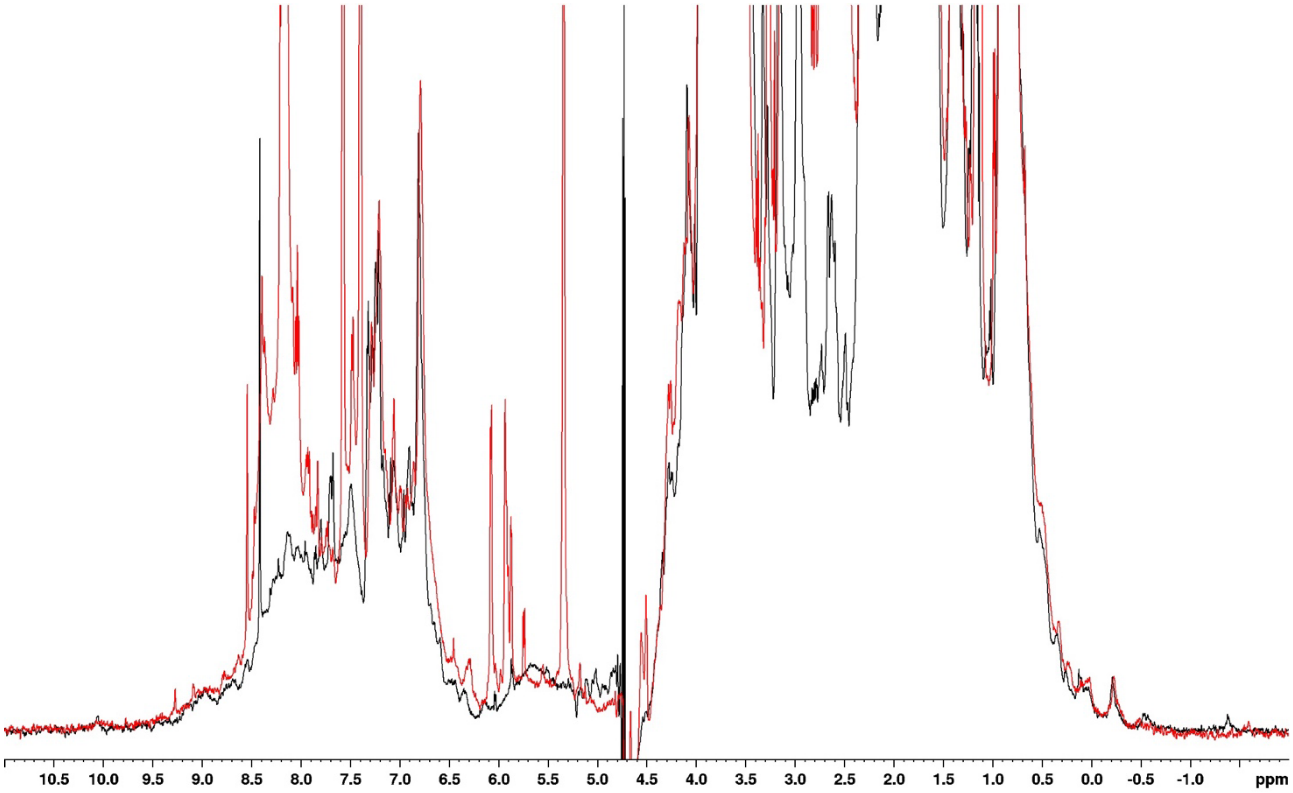

Figure S3. 1D ^{1}H-NMR spectra of $KHSRP_{fl}$ (20 μM) in 50 mM Tris-HCl buffer, pH 7.4, containing 500 mM NaCl, acquired at 25 °C. The protein was purified using the tandem method (black line) and the GST affinity chromatography only (red line).

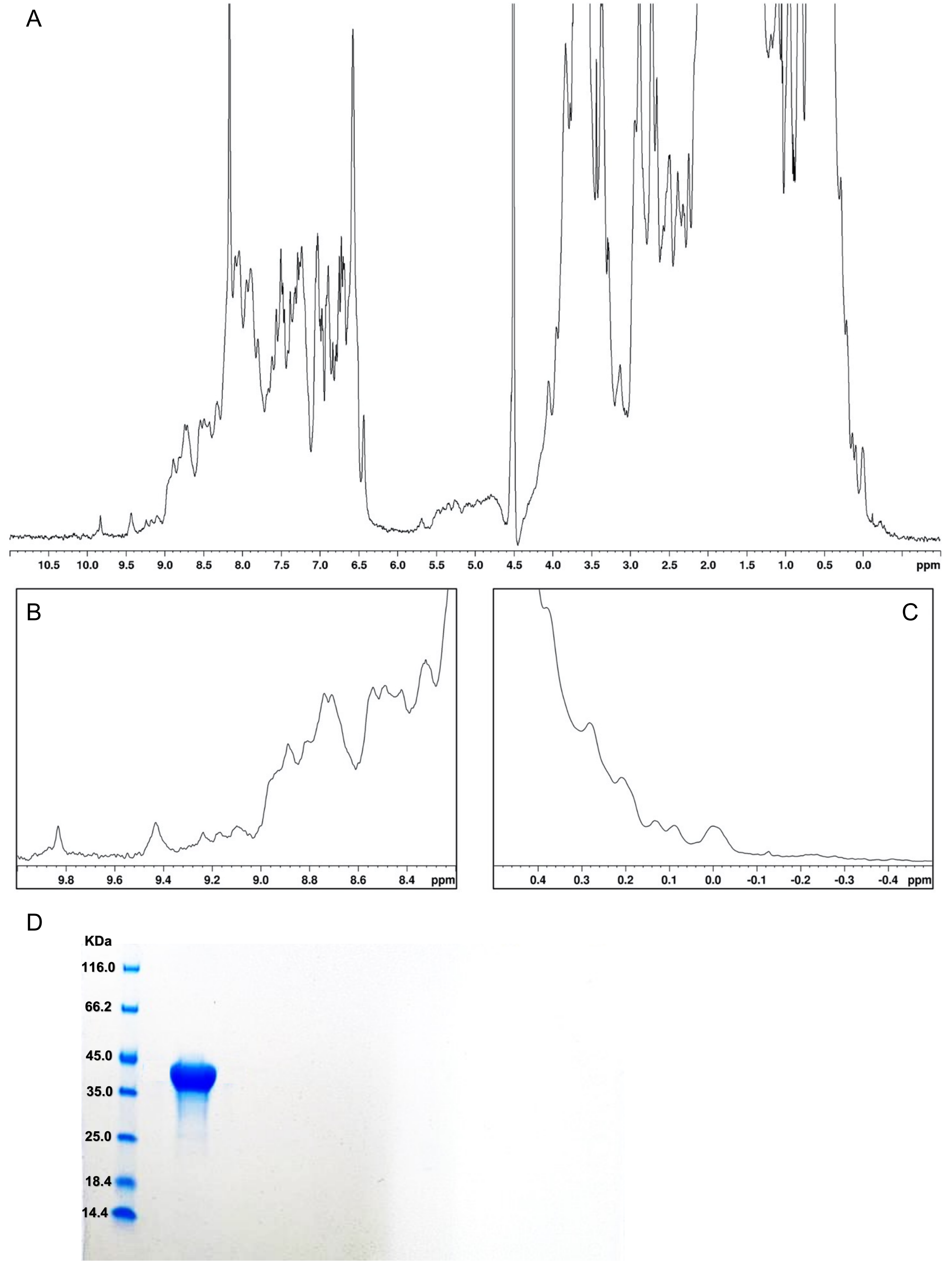

Figure S4. (A) 1D ^{1}H-NMR spectrum of $KHSRP_{130\text{-}503}$ (20 μM) in 5 mM KH_2PO_4/K_2HPO_4 buffer, pH 7.0, containing 20 mM KCl, acquired at 25 °C. The insets show the (B) aromatic and (C) aliphatic regions diagnostic of the protein folding. (D) SDS-PAGE of purified $KHSRP_{130\text{-}503}$.

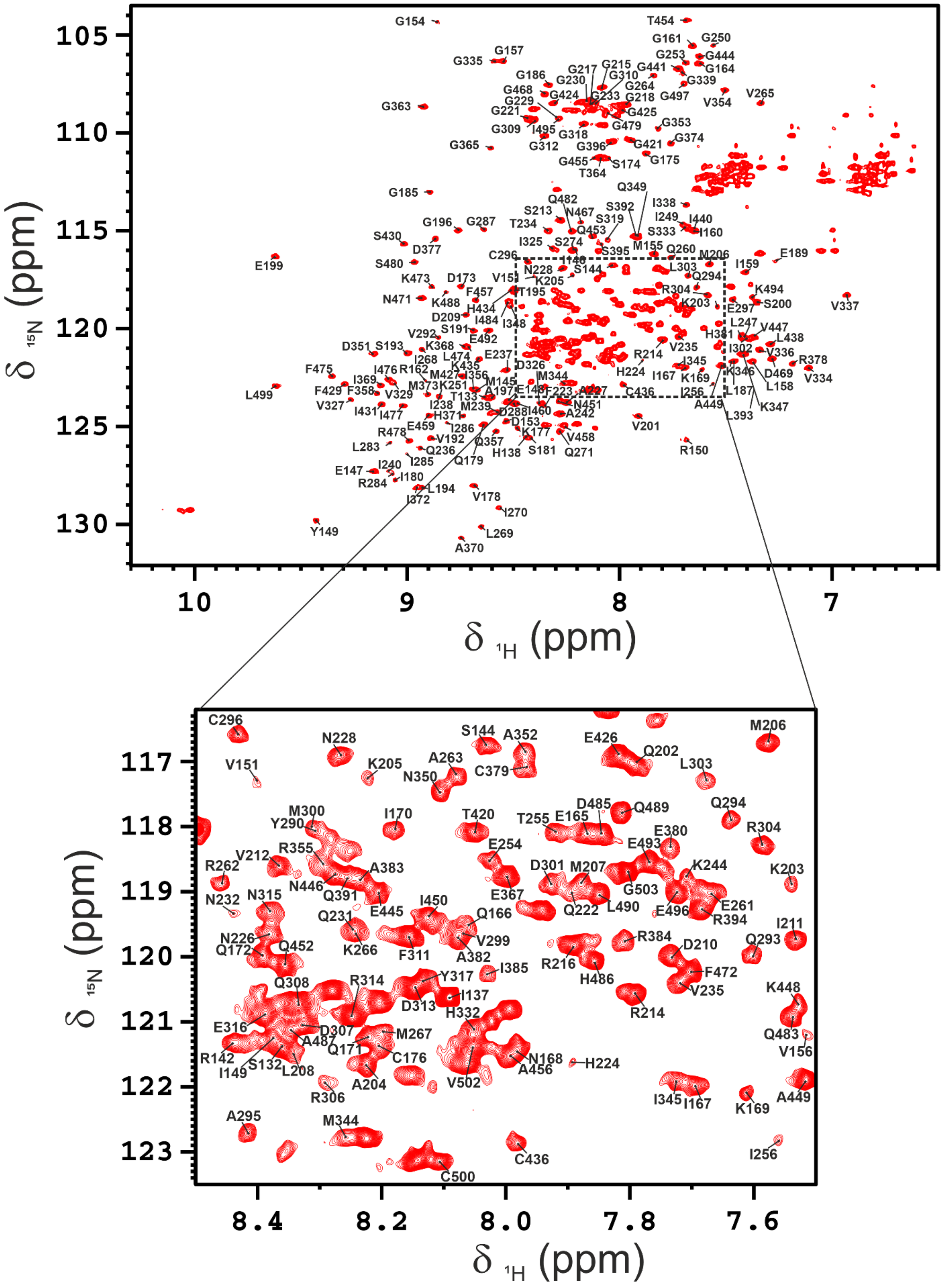

Figure S5. 2D ^{1}H ^{15}N HSQC of KHSRP$_{130\text{-}503}$ acquired on a spectrometer operating at 1200 MHz and 25 °C with the assignment reported on the signals.

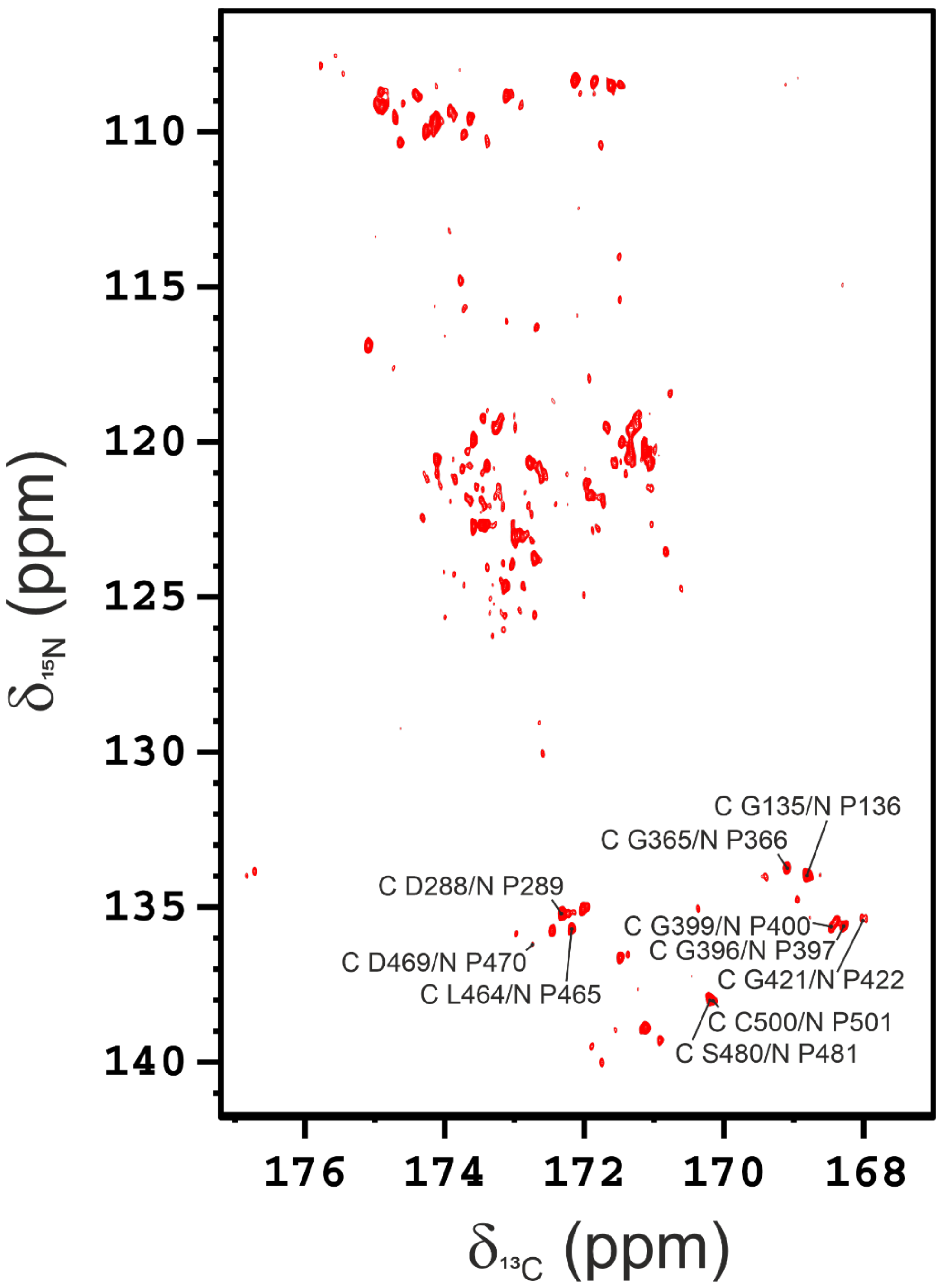

Figure S6. 2D ^{13}C-^{15}N (Hα)CON spectrum of free $KHSRP_{130-503}$. The assignment is reported for some identified proline residues. The spectrum was recorded on a spectrometer operating at 1200 MHz, ^{1}H Larmor frequency, and 25 °C.

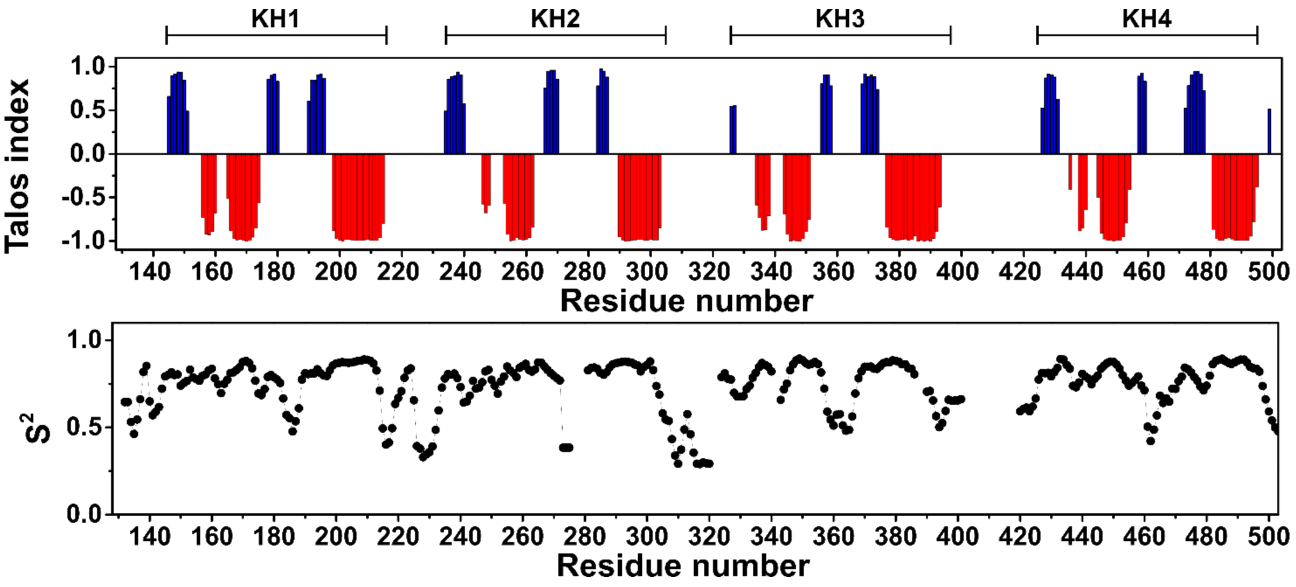

Figure S7. (Top) Secondary structure prediction obtained by the program Talos+ using the experimental values of chemical shifts of HN, N, C', Cα, and Cβ atoms as input data. The blue bars indicate the β-strand propensity while the red bars the α-helix propensity. (Bottom) Predicted order parameter (S^2) by the program Talos+ using the experimental values of chemical shifts of HN, N, C', Cα, and Cβ atoms as input data.

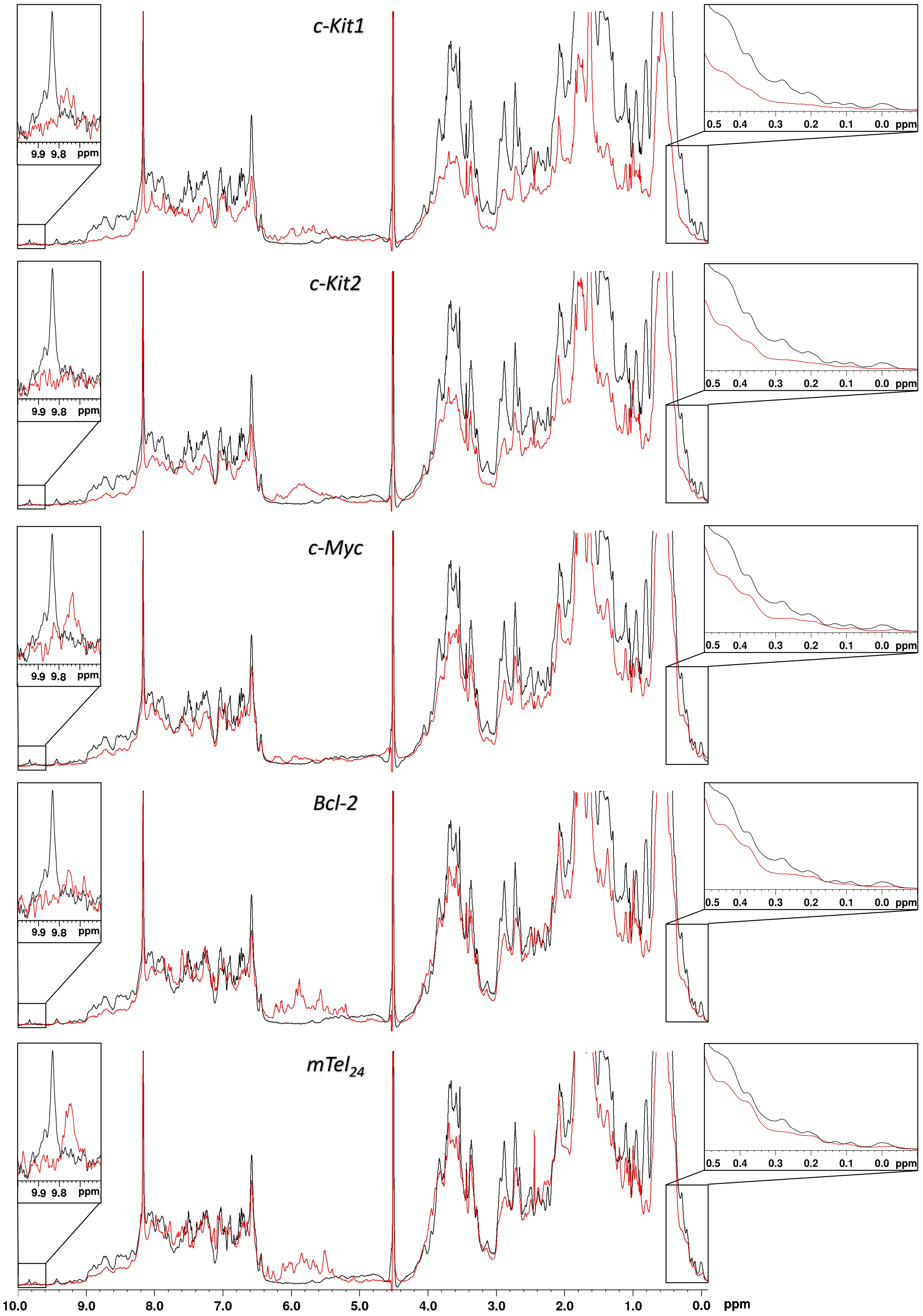

Figure S8. 1D ^{1}H-NMR spectra of $KHSRP_{130\text{-}503}$ (20 μM) in the absence (black line) and presence (red line) of G4s (1:1 ratio), acquired at 25 °C. The insets on the left and right show the tryptophan and aliphatic regions of the protein, respectively, used to monitor protein NMR signals in the absence and presence of DNA molecules.

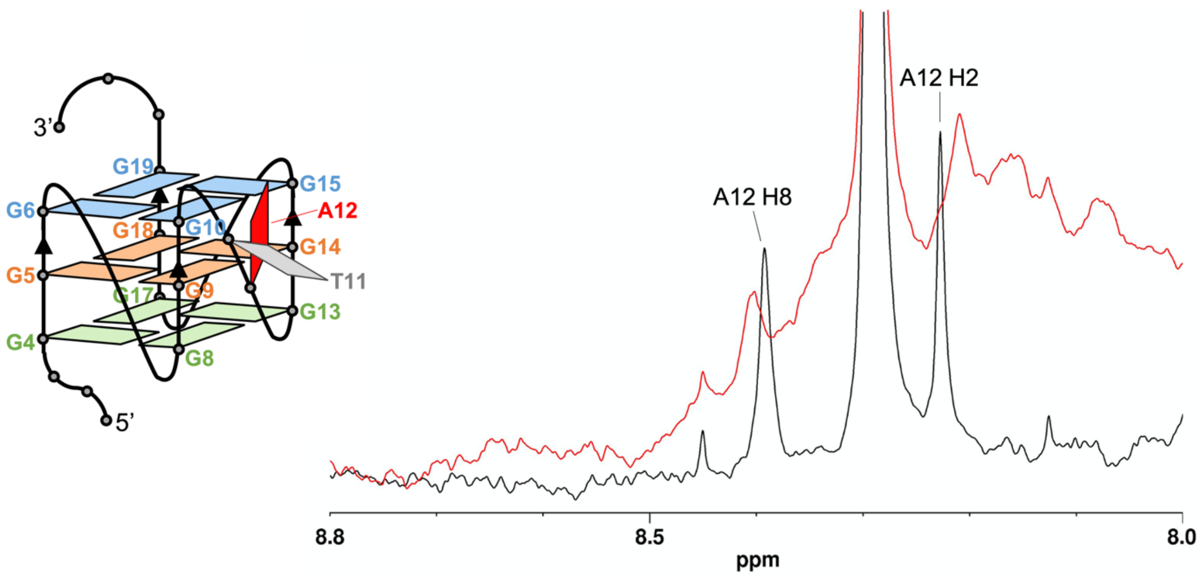

Figure S9. (Left) Schematic representation of the *c-Myc* G4. The guanines forming the G-tetrads are depicted as blue (3’ G-tetrad), orange (middle G-tetrad), and green (5’ G-tetrad) boxes. Thymine T11 and adenine A12 belonging to the double nucleotide loop are depicted as gray and red boxes, respectively. Strand directionalities are indicated by arrows. (Right) Aromatic proton region of *c-Myc* G4 (20 μM) in the absence (black line) and presence (red line) of KHSRP$_{130\text{-}503}$ protein acquired at 25 °C.

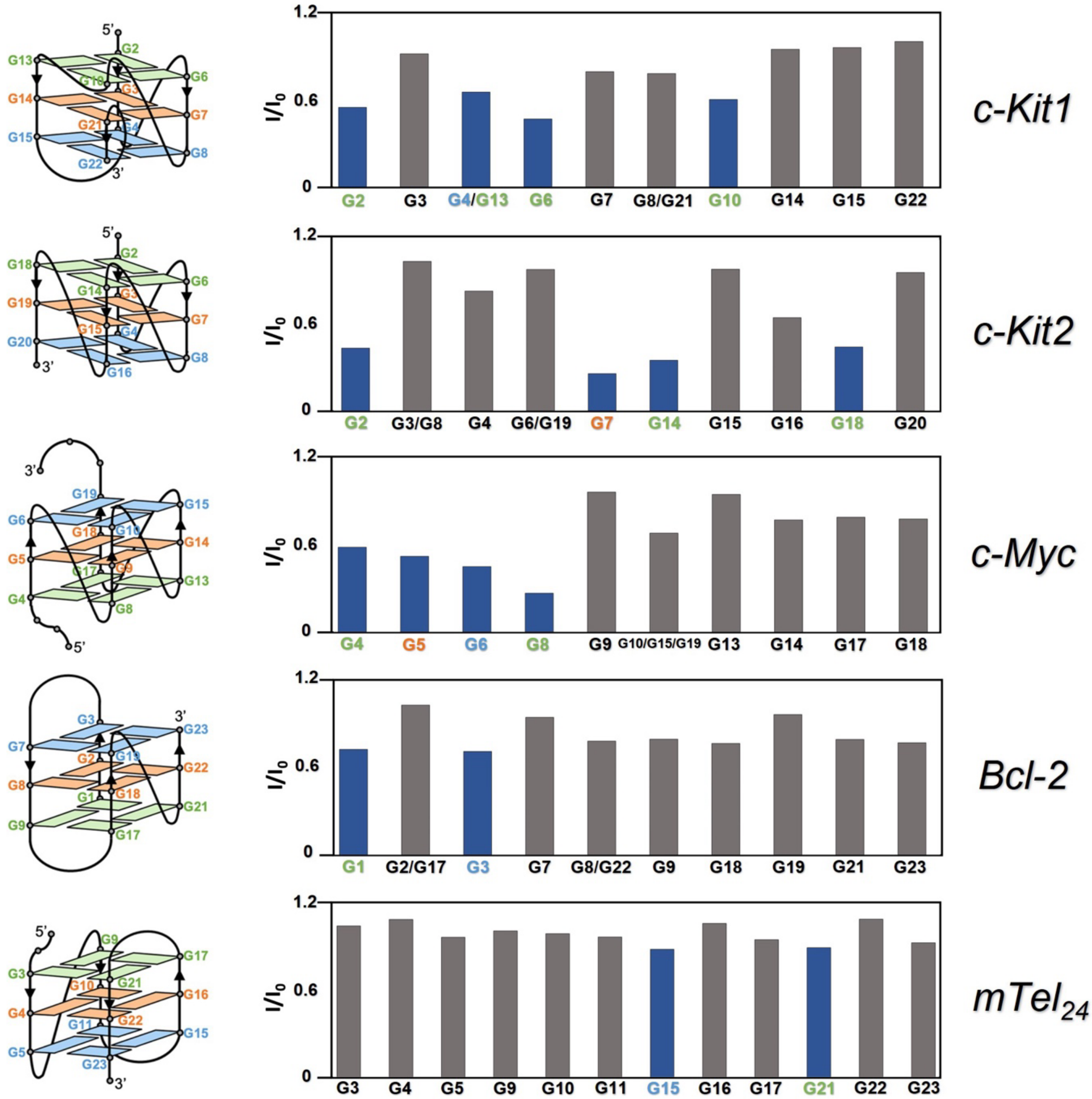

Figure S10. Relative intensities of the signals of the imino protons in 1D ^{1}H NMR spectra of G4s in the presence of KHSRP$_{130\text{-}503}$ with respect to the free G4s. In each spectrum, the intensities of the signals of the imino protons were normalized relative to the intensity of an isolated signal unaffected by the addition of KHSRP$_{130\text{-}503}$. Normalization was performed on the signal of G3 for *c-Kit1*, G15 for *c-Kit2*, G9 for *c-Myc*, G7 for *Bcl-2*, and G5 for *mTel*$_{24}$, respectively. The normalized intensities of the signals in the spectra of G4s in the presence of KHSRP$_{130\text{-}503}$ were compared to the normalized intensities of the same signals in the absence of the protein. The residues most affected by the interaction with KHSRP$_{130\text{-}503}$ are highlighted in blue in the plot.

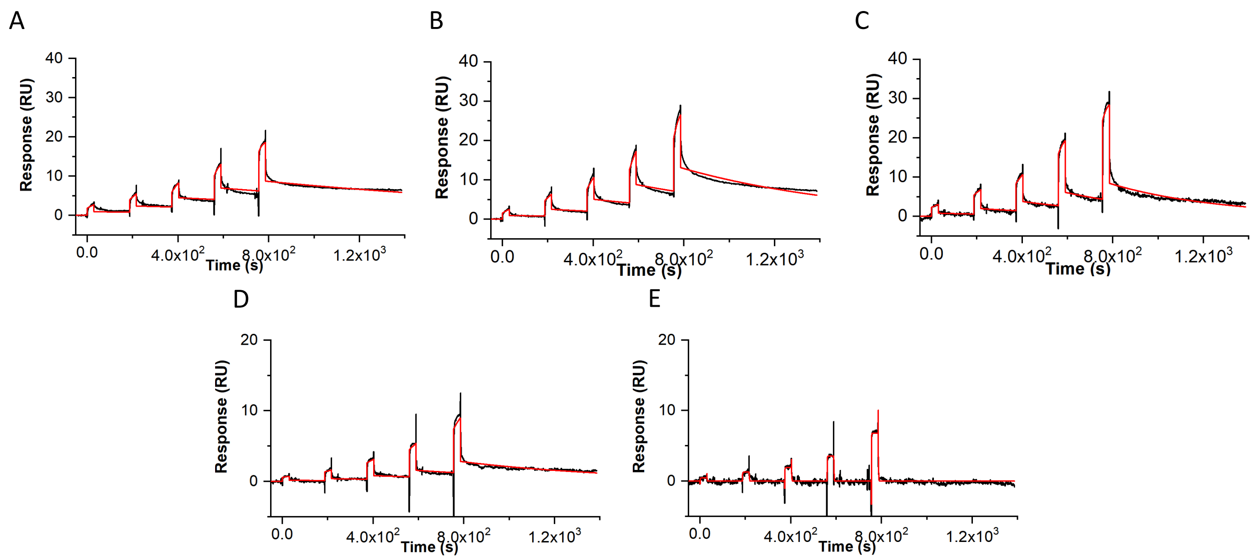

Figure S11. Representative replicates of SPR sensorgrams obtained at 25 °C by injections of various concentrations (from 1 to 20 μM) of (A) *c-Kit1*, (B) *c-Kit2*, (C) *c-Myc*, (D) *Bcl-2*, and (E) *mTel$_{24}$* G4s on the chip-immobilized KHSRP$_{130\text{-}503}$, with a contact time of 30 s and a flow rate of 30 μL/min. The sensorgrams are shown as black lines and their respective fits as red lines.

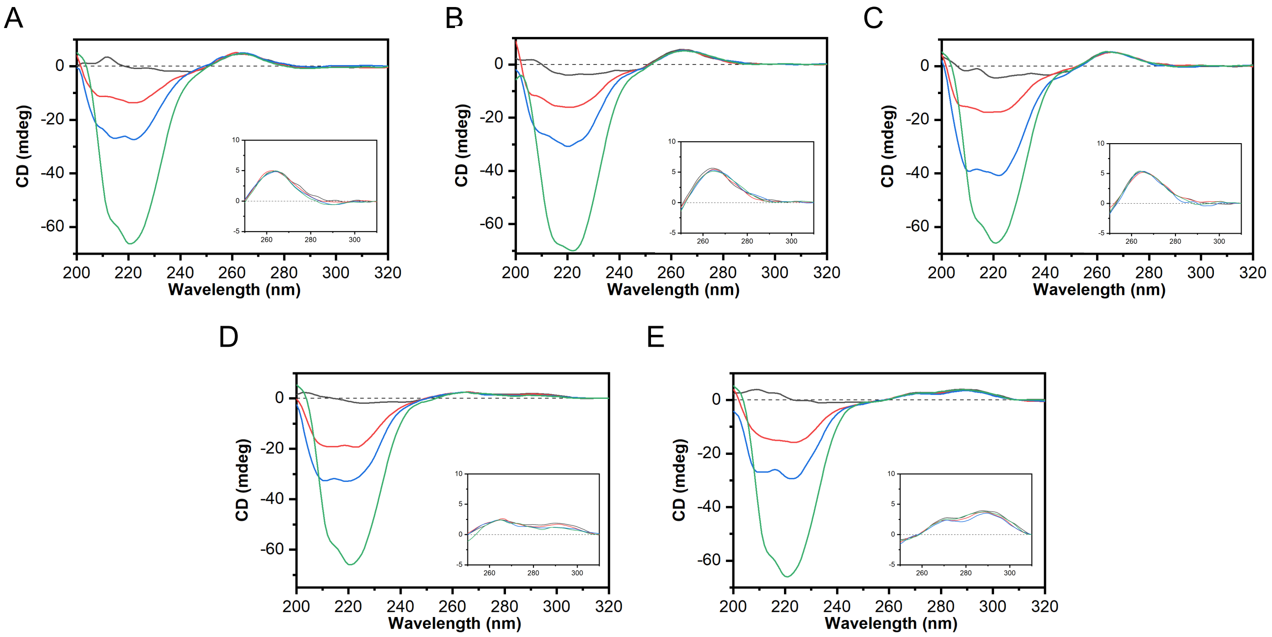

Figure S12. CD spectra of (A) *c-Kit1*, (B) *c-Kit2*, (C) *c-Myc*, (D) *Bcl-2*, and (E) *mTel$_{24}$* G4s (5 µM) at 25 °C in the absence (black lines) and presence of 1 (red lines), 2 (blue lines), and 4 (green lines) molar equiv. of KHSRP$_{130\text{-}503}$. Insets show the spectral region above 245 nm where only the G4 chromophores absorb.

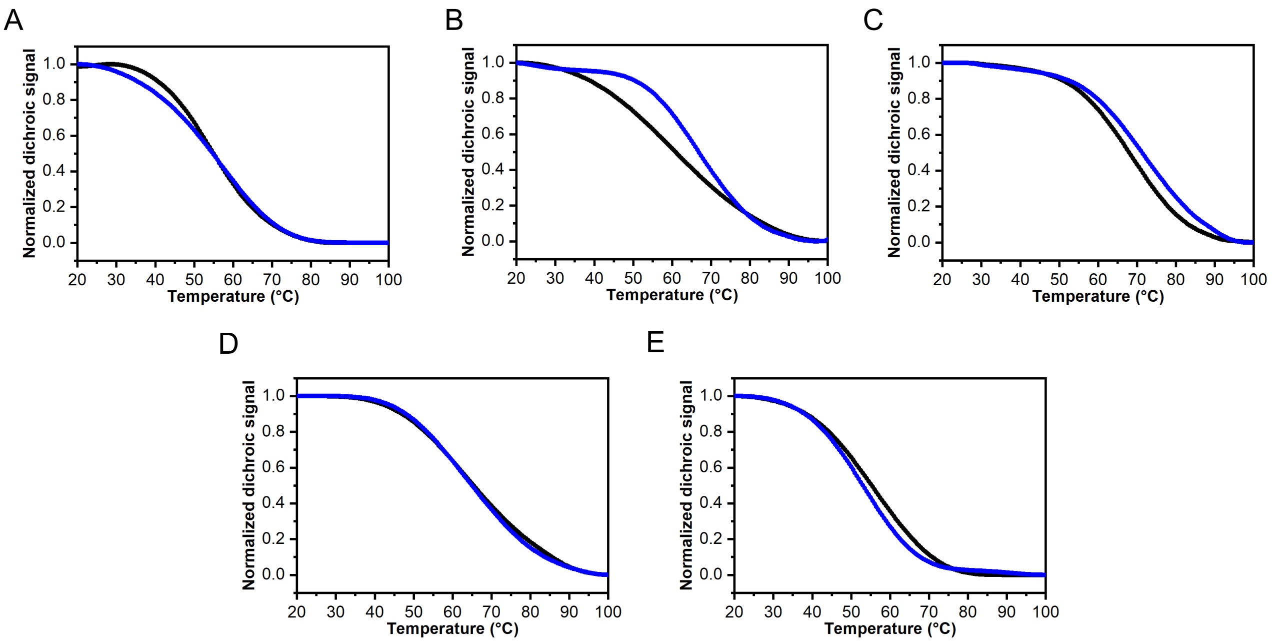

Figure S13. Normalized CD melting of (A) *c-Kit1*, (B) *c-Kit2*, (C) *c-Myc*, (D) *Bcl-2*, and (E) *mTel$_{24}$* G4s in the absence (black lines) and presence of equivalent amounts of KHSRP$_{130\text{-}503}$ (blue lines).

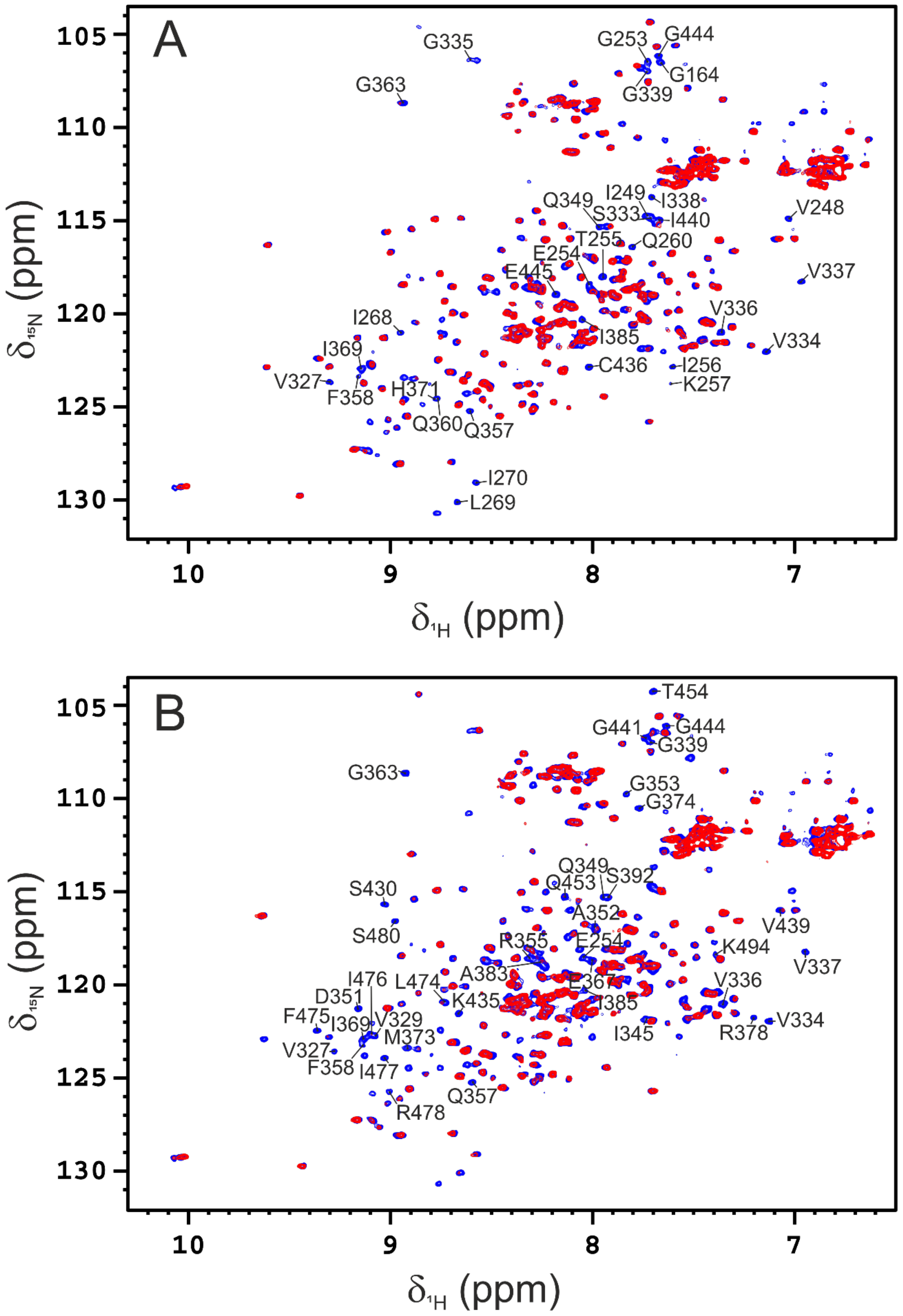

Figure S14. 2D ^{1}H-^{15}N HSQC spectra of free KHSRP$_{130\text{-}503}$ (50 µmol·dm^{-3}, blue) and KHSRP$_{130\text{-}503}$ in the presence of 25 µmol·dm^{-3} *c-Myc* (red, A) and in the presence of 6.25 µmol·dm^{-3} *c-Kit2* (red, B). The spectra were acquired on a spectrometer operating at 900 MHz, ^{1}H Larmor frequency, and 25 °C. Assignment of the signals experiencing the largest decreases in intensity is reported in the figure.

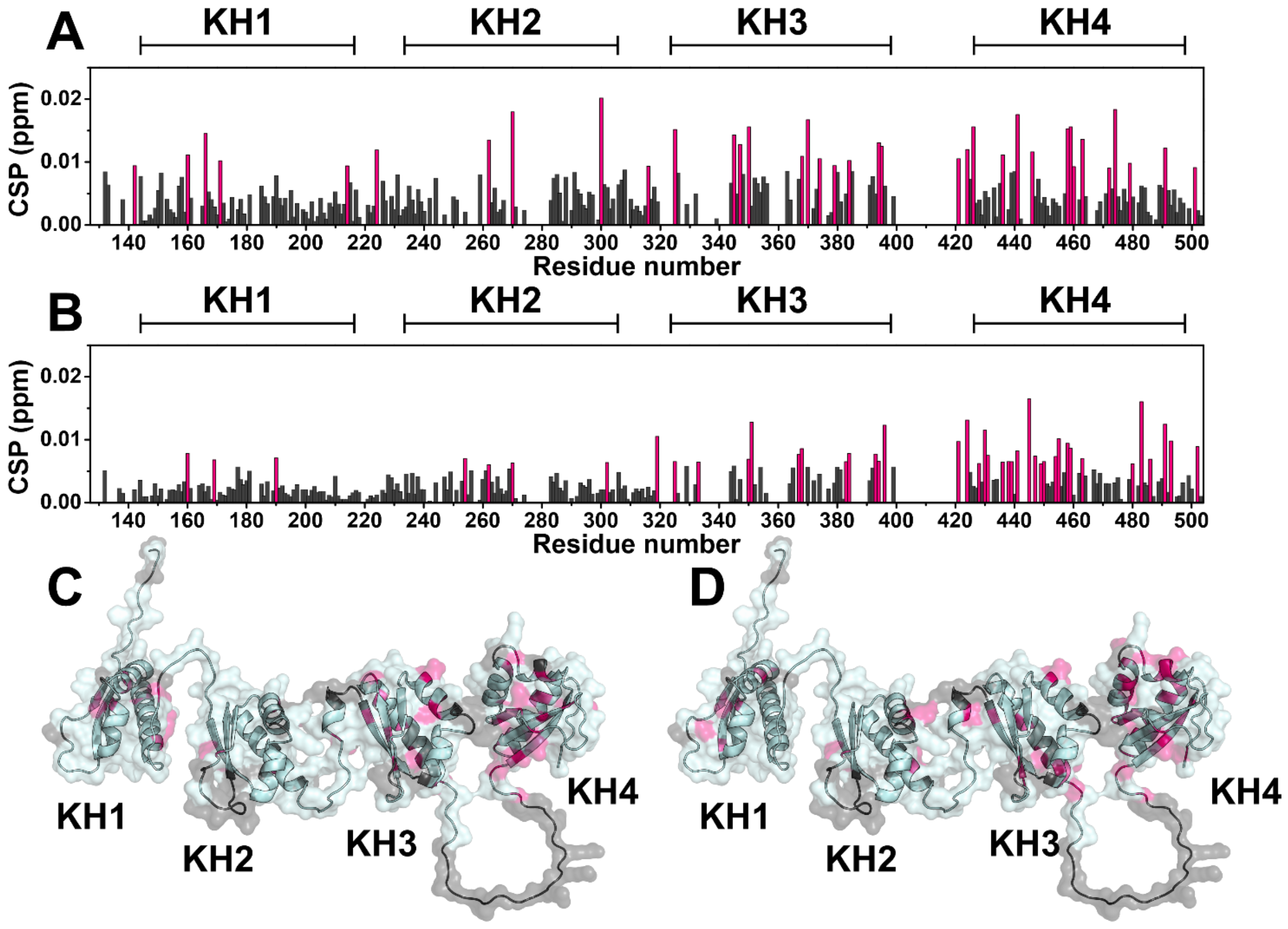

Figure S15. Graphical representation of the per-residue chemical shift perturbations of KHSRP (50 μM) in the presence of 25 μM concentration of *c-Myc* (A,C) and 6.25 μM of *c-Kit2* (B,D), evaluated with the formula $\Delta\delta = \frac{1}{2}\sqrt{\Delta\delta_H^2 + (\Delta\delta_N/5)^2}$. The residues exhibiting the largest CSP (*c-Myc*: Arg142, Ile160, Gln166, Gln171, Arg214, His224, Arg262, Ile270, Met300, Glu316, Ile325, Ile345, Lys347, Asn350, Lys368, Ala370, Gly374, Cys379, Arg384, Arg394, Ser395, Gly421, Gly424, Glu426, Cys436, Gly441, Asn446, Val458, Glu459, Ile460, Gln463, Phe472, Leu474, Gly479, Ile491; *c-Kit2*: Ile160, Lys169, Arg190, Glu254, Arg262, Ile270, Ile302, Ser319, Ile325, Ser333, Asn350, Asp351, Glu367, Lys368, Ala383, Arg384, Leu393, Arg394, Gly396, Gly421, Gly424, Thr428, Ser430, Ile431, Cys436, Leu438, Val439, Gly441, Glu445, Val447, Ala449, Ile450, Thr454, Gly455, Val458, Glu459, Gln463, Ser480, Gln483, His486, Ile491, Glu493, Val502) are colored in magenta in the plots (A,B) and on the AlphaFold model of the protein (C,D). Residues that could not be assigned in the NMR spectra are colored in gray on the protein model.

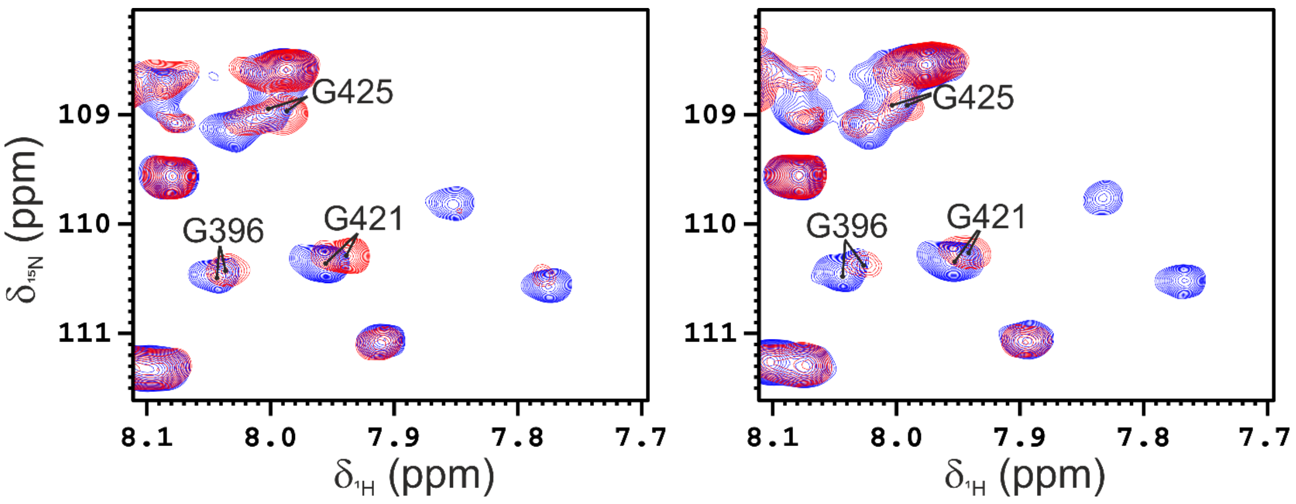

Figure S16. Portions of 2D ^{1}H-^{15}N HSQC spectra of free $KHSRP_{130\text{-}503}$ (50 µM, blue) and $KHSRP_{130\text{-}503}$ in the presence of 25 $\mu mol \cdot dm^{-3}$ *c-Myc* (red, left) and in the presence of 6.25 µM *c-Kit2* (red, right), showing some signals assigned to residues of the linker undergoing changes in their chemical shift. The spectra were acquired on a spectrometer operating at 900 MHz, ^{1}H Larmor frequency, and 25 °C.

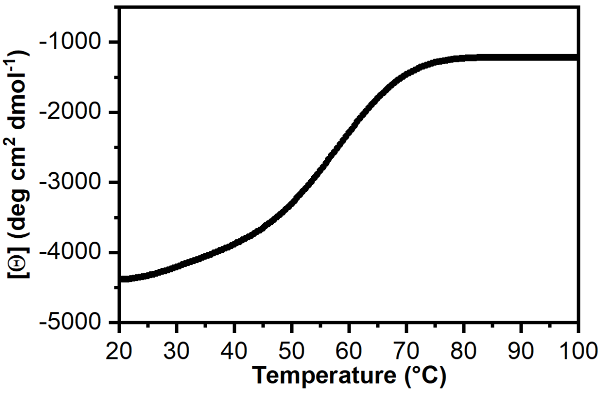

Figure S17. Thermal unfolding curve of KHSRP$_{130-503}$ obtained by recording the molar ellipticity at 222 nm using 1.0 °C/min scan rate.

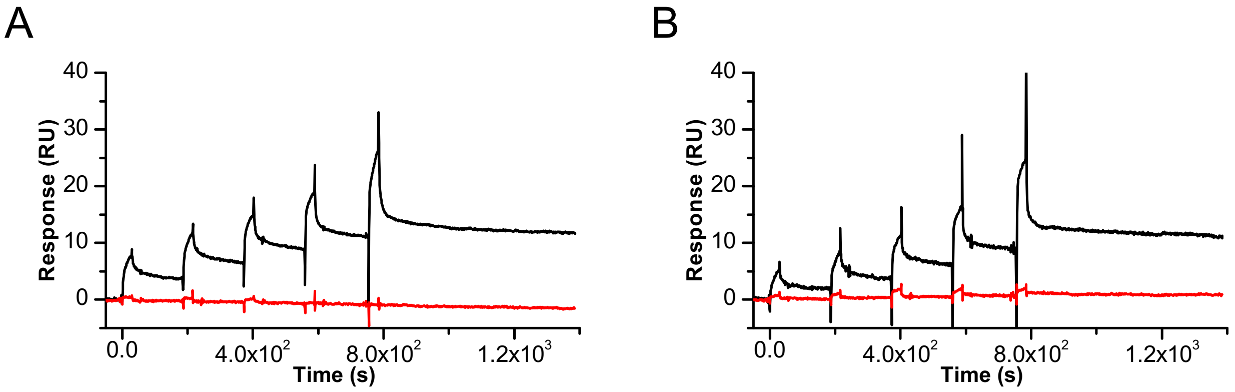

Figure S18. Superimposed SPR sensorgrams obtained at 25 °C by injections of various concentrations (from 1 to 20 μM) of (A) *c-Kit2* and (B) *c-Myc* G4s in the absence (black line) and presence (red line) of 2 molar equiv of pyridostatin on the chip-immobilized KHSRP$_{130-503}$, with a contact time of 30 s and a flow rate of 30 μL/min.

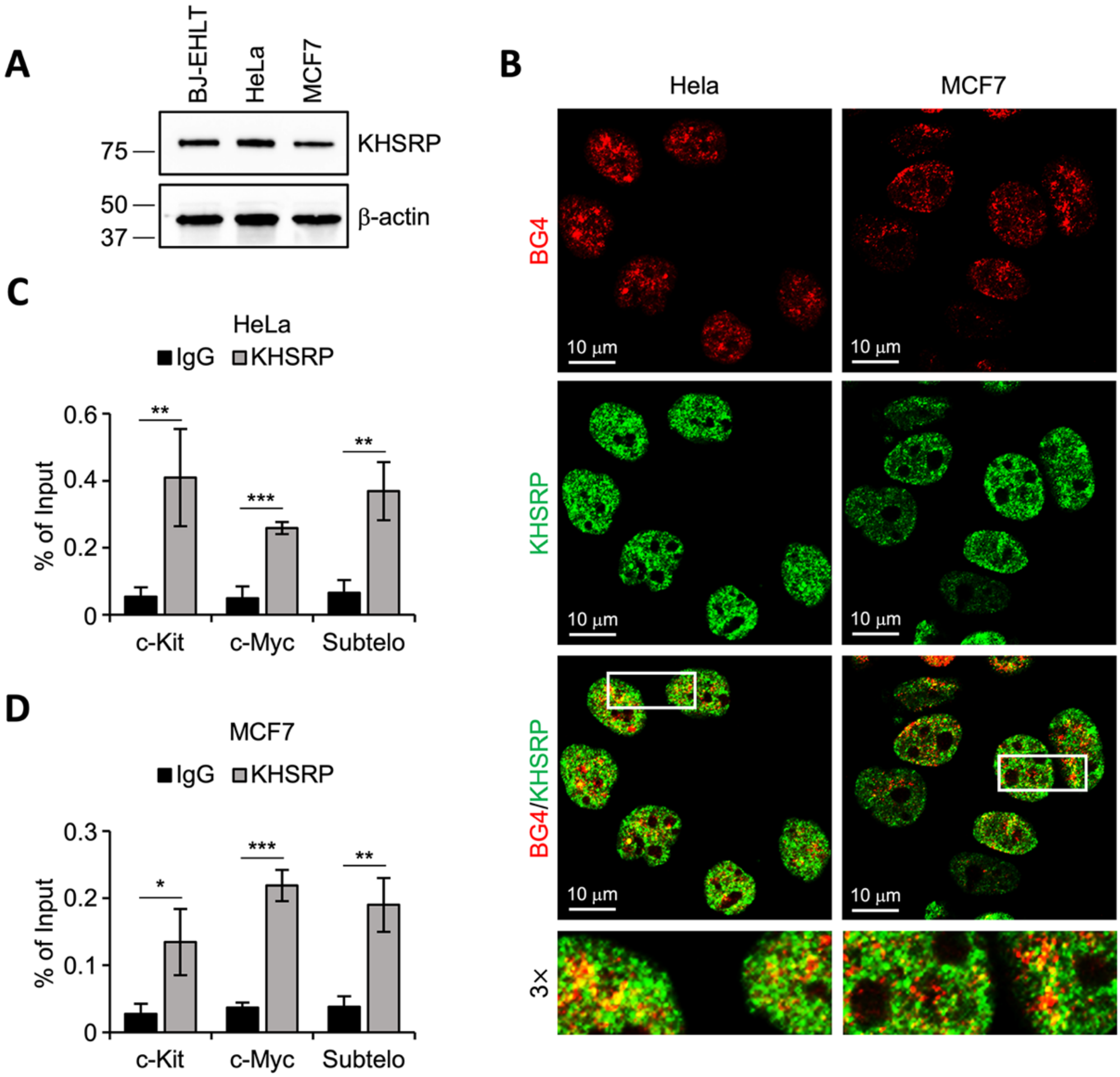

Figure S19. KHSRP localizes in G4 forming regions of *c-KIT* and *c-MYC* promoters independently of tumor histotype. (A) Western blot analysis showing the expression levels of KHSRP in BJ-EHLT, HeLa, and MCF-7 cell lines. β-actin was used as internal loading control. (B) Representative images of immunofluorescence (IF) analyses performed in cervical carcinoma (HeLa) and a breast adenocarcinoma (MCF-7) cell lines, using the antibodies against G4s (anti-BG4, red spots) and KHSRP (green spots). Specific enlargements (3×) are shown. The images were acquired by confocal microscopy (magnification 63×). Scale bar: 10 µm. (C,D) Chromatin Immunoprecipitation (ChIP) assays performed in HeLa and MCF-7 cells by immunoprecipitating protein-DNA complex with the antibody against KHSRP. Rabbit IgG was used as negative control. KHSRP enrichment at G4-forming sequence of the promoter of *c-KIT* and *c-MYC* and at subtelomeric regions was evaluated by qPCR Real Time analyses. The KHSRP enrichment was expressed as percentage of input. The histograms represent the mean values ± S.D. of three independent experiments. *$p<0.05$, **$p<0.01$, ***$p<0.001$.

Table S1. List of qPCR-ChIP primers.

Target Region	Sequence (5'→3')
Sub-Telo Chr 2 Forward	CCCAAACCCTAACCCTAAAA
Sub-Telo Chr 2 Reverse	CTTCCTGTTTGCAGCACTGA
c-Myc Promoter (prom Myc) Forward	GCGGAGATTAGCGAGAGAGG
c-Myc Promoter (prom Myc) Reverse	TGCGGCTCTCTTACTCTGTT
c-Kit Promoter (prom Kit) Forward	CCGGGAAGAAGCGAGACC
c-Kit Promoter (prom Kit) Reverse	GGTAGCTGCGATGGGATCC

References

[1] S. Pelliccia, J. Amato, D. Capasso, S. Di Gaetano, A. Massarotti, M. Piccolo, C. Irace, G. C. G. C. Tron, B. Pagano, A. Randazzo, E. Novellino, M. Giustiniano, *J Med Chem* **2020**, *63*, 2035.
[2] A. T. Phan, V. Kuryavyi, S. Burge, S. Neidle, D. J. Patel, *J Am Chem Soc* **2007**, *129*, 4386.
[3] V. Kuryavyi, A. T. Phan, D. J. Patel, *Nucleic Acids Res* **2010**, *38*, 6757.
[4] A. Ambrus, D. Chen, J. Dai, R. A. Jones, D. Yang, *Biochemistry* **2005**, *44*, 2048.
[5] J. Dai, D. Chen, R. A. Jones, L. H. Hurley, D. Yang, *Nucleic Acids Res* **2006**, *34*, 5133.
[6] K. N. Luu, A. T. Phan, V. Kuryavyi, L. Lacroix, D. J. Patel, *J Am Chem Soc* **2006**, *128*, 9963.
[7] H. Cantor, C. R.; Warshaw, M. M.; Shapiro, C. R. Cantor, M. M. Warshaw, H. Shapiro, *Biopolymers* **1970**, *9*, 1059.
[8] T. L. Hwang, A. J. Shaka, *J Magn Reson A* **1995**, *112*, 275.
[9] S. Grzesiek, A. Bax, *Journal of Magnetic Resonance (1969)* **1992**, *96*, 432.
[10] J. Schleucher, M. Sattler, C. Griesinger, *Angewandte Chemie International Edition in English* **1993**, *32*, 1489.
[11] L. E. Kay, G. Y. Xu, T. Yamazaki, *J Magn Reson A* **1994**, *109*, 129.
[12] M. Bostock, D. Nietlispach, *Concepts in Magnetic Resonance Part A* **2017**, *46A*.
[13] W. Bermel, I. Bertini, I. C. Felli, Y.-M. Lee, C. Luchinat, R. Pierattelli, *J Am Chem Soc* **2006**, *128*, 3918.
[14] W. Bermel, I. Bertini, L. Duma, I. C. Felli, L. Emsley, R. Pierattelli, P. R. Vasos, *Angewandte Chemie International Edition* **2005**, *44*, 3089.
[15] R. Keller, *The computer aided resonance assignment tutorial*, **2004**.
[16] Y. Shen, F. Delaglio, G. Cornilescu, A. Bax, *J Biomol NMR* **2009**, *44*, 213.
[17] R. Karlsson, P. S. Katsamba, H. Nordin, E. Pol, D. G. Myszka, *Anal Biochem* **2006**, *349*, 136.
[18] C. Di Primo, *Journal of Molecular Recognition* **2008**, *21*, 37.
[19] W. Palau, C. Di Primo, *Biochimie* **2012**, *94*, 1891.
[20] P. L. Privalov, S. A. Potekhin, *Methods Enzymol* **1986**, *131*, 4.
[21] W. Chen, W. C. Hahn, *Histol Histopathol* **2003**, *18*, 541.
[22] R. Dinami, L. Pompili, E. Petti, M. Porru, C. D'Angelo, S. Di Vito, A. Rizzo, V. Campani, G. De Rosa, A. Bruna, V. Serra, M. Mano, M. Giacca, C. Leonetti, G. Ciliberto, M. Tarsounas, A. Stoppacciaro, S. Schoeftner, A. Biroccio, *EMBO Mol Med* **2023**, *15*, e16033.
[23] S. Iachettini, D. Trisciuoglio, D. Rotili, A. Lucidi, E. Salvati, P. Zizza, L. Di Leo, D. Del Bufalo, M. R. Ciriolo, C. Leonetti, C. Steegborn, A. Mai, A. Rizzo, A. Biroccio, *Cell Death Dis* **2018**, *9*, 996.
